# Light‐Controlled Electric Stimulation with Organic Electrolytic Photocapacitors Achieves Complex Neuronal Network Activation: Semi‐Chronic Study in Cortical Cell Culture and Rat Model

**DOI:** 10.1002/adhm.202401303

**Published:** 2024-08-13

**Authors:** Marta Nowakowska, Marie Jakešová, Tony Schmidt, Aleksandar Opančar, Mathias Polz, Robert Reimer, Julia Fuchs, Silke Patz, Daniel Ziesel, Susanne Scheruebel, Karin Kornmueller, Theresa Rienmüller, Vedran Đerek, Eric D. Głowacki, Rainer Schindl, Muammer Üçal

**Affiliations:** ^1^ Department of Neurosurgery Medical University of Graz Auenbruggerplatz 29 Graz 8036 Austria; ^2^ BioTechMed‐Graz Mozartgasse 12/II Graz 8010 Austria; ^3^ Bioelectronics Materials and Devices Laboratory CEITEC Brno University of Technology Purkyňova 123 Brno 612 00 Czech Republic; ^4^ Gottfried Schatz Research Center for Cell Signaling Metabolism and Aging Division of Medical Physics and Biophysics Medical University of Graz Neue Stiftingtalstraße 6 Graz 8010 Austria; ^5^ Department of Physics Faculty of Science University of Zagreb Bijenička c. 32 Zagreb 10000 Croatia; ^6^ Institute of Health Care Engineering with European Testing Center of Medical Devices Graz University of Technology Stremayrgasse 16/II Graz 8010 Austria; ^7^ Department of Neurology Medical University of Graz Auenbruggerplatz 22 Graz 8036 Austria

**Keywords:** bioelectronics, cortical stimulation, c‐Fos, excitability, immunohistochemistry, organic semiconductors, wireless stimulation

## Abstract

Neurostimulation employing photoactive organic semiconductors offers an appealing alternative to conventional techniques, enabling targeted action and wireless control through light. In this study, organic electrolytic photocapacitors (OEPC) are employed to investigate the effects of light‐controlled electric stimulation on neuronal networks in vitro and in vivo. The interactions between the devices and biological systems are characterized. Stimulation of primary rat cortical neurons results in an elevated expression of c‐Fos within a mature neuronal network. OEPC implantation for three weeks and subsequent stimulation of the somatosensory cortex leads to an increase of c‐Fos in neurons at the stimulation site and in connected brain regions (entorhinal cortex, hippocampus), both in the ipsi‐ and contralateral hemispheres. Reactivity of glial and immune cells after semi‐chronic implantation of OEPC in the rat brain is comparable to that of surgical controls, indicating minimal foreign body response. Device functionality is further substantiated through retained charging dynamics following explantation. OEPC‐based, light‐controlled electric stimulation has a significant impact on neural responsiveness. The absence of detrimental effects on both the brain and device encourages further use of OEPC as cortical implants. These findings highlight its potential as a novel mode of neurostimulation and instigate further exploration into applications in fundamental neuroscience.

## Graphical abstract

Wireless cortical stimulation is an attractive alternative to standard neurostimulation methods. Organic photoactive semiconductors can transduce light into electric signal, even in ultrathin layers, which can be used to stimulate neurons. In this study, light‐based stimulation activated complex neuronal networks in vitro and in vivo, including deep brain regions. The devices proved safe and stable over three weeks of implantation.

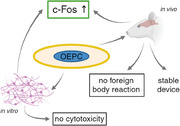

## 1 Introduction

The application of exogenous electric current modulates the activity of excitable nervous tissue in a complex manner, which can be leveraged to treat various neurological disorders. Electric neurostimulation, as a therapeutic method, gains a wide‐ranging applicability, having proven effective in the treatment of conditions such as Parkinson's disease and other movement disorders,^[^ ^1^, ^2^ ^]^ refractory epilepsy,^[^ ^3^, ^4^ ^]^ chronic pain^[^ ^5^, ^6^, ^7^, ^8^ ^]^ and disorders of mood.^[^ ^9^, ^10^ ^]^ In the central nervous system, electric stimulation exerts diverse effects: it enhances neuronal plasticity,^[^ ^11^ ^]^ promotes axonal outgrowth post‐injury,^[^ ^12^, ^13^ ^]^ and up‐regulates expression of neurotrophins^[^ ^12^, ^14^, ^15^ ^]^ and growth‐associated factors.^[^ ^12^ ^]^ Cumulatively, these effects contribute to an overall improvement in functional recovery following injury.^[^ ^16^, ^17^, ^18^, ^19^ ^]^ The capacity to influence the nervous system highlights the potential of electric neurostimulation as a potent and widely applicable therapy with substantial benefits. Amongst the available methods, wireless stimulation devices pose an attractive alternative to the existing methods.^[^ ^20^ ^]^

The use of photoactive materials for wireless neurostimulation has been under investigation for the past two decades.^[^ ^21^, ^22^ ^]^ Substantial progress in the field was made in the 2010s, particularly in the development of photovoltaic implants for retina stimulation.^[^ ^23^, ^24^, ^25^ ^]^ Light‐controlled electric stimulation offers numerous advantages that can be harnessed for effective modulation of the nervous tissue. It provides a convenient and wireless control mechanism using light, which can be easily adjusted for intensity, frequency, and pulse duration. The use of light in the red spectrum allows for a tissue penetration up to 13 mm,^[^ ^26^, ^27^ ^]^ enabling transdermal stimulation. Effective stimulation currents can be generated in thin layers (<100 nm) of organic photosensitive materials.^[^ ^28^ ^]^ When processed on ultrathin plastic foils (1–10 µm thickness), remarkably lightweight and compact photostimulation devices can be manufactured. The combination of miniaturization and wireless control could lead to minimally invasive implantation, reducing the risk of infection associated with externalized stimulators and leads.^[^ ^29^, ^30^ ^]^ Moreover, the use of common non‐toxic organic photoactive pigments, already employed in cosmetics and medical products, simplifies device manufacturing without compromising biocompatibility and safety.^[^ ^31^ ^]^

Photoactive semiconductor devices typically consist of a combination of electron‐accepting (*n*‐type) and hole‐accepting (*p*‐type) components, forming a light‐sensitive *p–n* junction. Upon illumination, an exciton (a bound electron‐hole pair) is generated, electrons and holes are separated at the *p–n* interface and collected by the *p* and *n* materials respectively, thereby charging the surface of the device. The charged surface can interact with the electrolyte either through electron transfer from the device onto the electrolyte compounds (photofaradaic stimulation) or without electron transfer, through the movement of ions present in the electrolyte, creating an electric double layer (photocapacitive stimulation). The capacitive mechanism, involving rapid charging and discharging of the electrode–electrolyte double layer, without reduction‐oxidation reactions connected to faradaic stimulation, is generally more desirable during stimulation.^[^ ^32^ ^]^

We have previously demonstrated the successful application of wireless and ultrathin organic electrolytic photocapacitors (OEPC) as an effective stimulation method both in cellular models^[^ ^33^, ^34^, ^35^ ^]^ and in animal experiments.^[^ ^27^, ^36^, ^37^, ^38^ ^]^ The key innovative step in OEPC is using xerographic organic pigments, which have been used for decades in commercial products and have well‐documented stability and nontoxicity. In several in vitro models, OEPC were proven to achieve suprathreshold neurostimulation, including single‐cell oocyte models and explanted retina tissues. In the oocyte model, it was possible to verify that OEPC stimulation easily achieves extracellular current stimulation thresholds to activate voltage‐gated channels^[^ ^34^ ^]^. Electrophysiological measurements conducted in experiments on mouse primary hippocampal neurons further verified precise action potential firing during light illumination of the OEPC.^[^ ^35^ ^]^ During these in vitro experiments, the OEPC platform was evaluated for stability using aging tests in solution, as well as lifetime testing involving light‐pulse stress. Devices demonstrated stability over 27 million charge‐discharge cycles. Flexible OEPC, microfabricated on ultrathin parylene‐C foils, were implanted onto the sciatic nerve of rats, and demonstrated successful stimulation over the course of 100 days, delivering current densities up to 2 mA cm^−2^, at a photovoltage up to 350 mV.^[^ ^27^, ^33^, ^34^ ^]^ This chronic validation involved transmitting light through up to 15 mm of intervening tissue. This experiment showed not only that chronic in vivo stimulation was possible, but showcased the efficacy of tissue penetrating red light in a safe and reliable way. Subsequent work demonstrated the OEPC platform for stimulation of the mouse vagus nerve.^[^ ^38^ ^]^ Besides nerve activation, this study approached the issue of photothermal heating with computer simulations, indicating its safe levels in all investigated tissues. Both studies validate the approach of red light penetrating tissue without spurious photothermal effects, and the ability of implanted OEPC to produce suprathreshold stimulation currents. Building up on this prior work, the present study aimed first to provide a more comprehensive understanding of the long‐term OEPC cytotoxicity and the influence of light‐controlled electric stimulation on interconnected neurons, addressing the lingering uncertainty about the broader impact beyond individual cells, and including the influence of summation of sub‐threshold depolarizations.^[^ ^39^ ^]^

Expanding upon our knowledge from experiments on cells, it became evident that OEPC could elicit action potentials in the somatosensory cortex in mice.^[^ ^36^ ^]^ Notably, the chronic implantation of the OEPC in the form of a nerve cuff proved sustained device efficacy, remaining effective for up to 100 days post‐surgery.^[^ ^27^ ^]^ Despite these advancements, comprehensive data regarding the long‐term effects of the implantation on both the brain and the device were lacking. Consequently, the second objective of the present study was to delve into the consequences of semi‐chronic implantation of small (⌀ 5 mm) and thin (3 µm) OEPC on the somatosensory cortex in adult male rats. This exploration encompassed evaluating device efficiency, assessing the impact of cortical stimulation on the neuronal network, and investigating the foreign body response of the brain tissue.

## 2 Results

### 2.1 Primary Cortical Cell Survival on OEPC

Primary cortical cells were cultivated on glass/ITO‐OEPCs coated with different standard coating substances (Geltrex, PDL, PEI; Figure S1, Supporting Information). Visual inspection of the culture revealed the highest cell density on the Geltrex‐coated samples (Figure S1A,E, Supporting Information), both during the first and the second week. However, this coating is based on the extracellular matrix, with thickness reaching up to 20 µm,^[^ ^40^ ^]^ which could potentially have a negative impact on OEPC charging. PDL coating also yielded satisfactory cell density and intricate neuronal network throughout the experiment (Figure S1B,F, Supporting Information). Interestingly, cells seeded on OEPCs coated with PEI, a material frequently used as a coating, e.g. on multi‐electrode array chips,^[^ ^41^, ^42^, ^43^ ^]^ did not attach to the surface (Figure S1C,G, Supporting Information). Although cells managed to adhere to uncoated OEPCs (Figure S1D,H, Supporting Information), the neuronal network appeared to be less dense than those on the devices coated with Geltrex or PDL. Hence, PDL coating was employed in further experiments.

To ascertain biocompatibility of OEPC in the cell culture, we cultivated cells on PDL‐coated glass/Au‐devices and glass coverslips for two weeks (Figure S2A, Supporting Information). Cells attached and started developing complex networks already at DIV5, reaching further maturation on DIV10 to DIV14. The addition of a mitosis inhibitor in order to interfere with an overgrowth of glial cells led to a decreased cell density over time. The comparison between the cells growing on top of the *p–n* layer and those on the back electrode did not reveal any discernible differences between them (Figure S2B, Supporting Information).

Cytotoxic effects of surface materials of glass/Au‐OEPC were assessed by means of LDH assay. Supernatants were collected from cultures covering the entire device surface. Consequently, the results reflect the cytotoxicity of the whole OEPC, including the organic materials of the *p–n* layer and the gold back electrode. LDH activity in the cell culture media did not differ between OEPC and glass (Figure S2C, Supporting Information; F(1, 8)_Group_  =  0.00042, *p* =  0.984). However, it significantly changed during the course of culture (F(1.16, 9.31)_Time_  =  86.82, *p* < 0.001) in both groups, increasing at DIV10 (*p* < 0.001) and decreasing at DIV14 (*p* < 0.001), attributable to glial cell death upon mitosis inhibition and maturation, respectively. LDH activity at DIV14 remained significantly higher than at DIV5 (*p*  =  0.0015), suggesting an ongoing selection against non‐neuronal cells and culture aging. Interestingly, LDH levels after 30 min 20 Hz light treatment exhibited a significant decrease compared to the levels prior to stimulation (Figure S2D, Supporting Information t(6.4038)  =  10.083, *p* < 0.001).

### 2.2 c‐Fos Expression Following Light Stimulation in Cell Culture

*c‐fos* is an immediate early gene that is rapidly and transiently activated following various stimuli.^[^ ^44^, ^45^ ^]^ In neuronal cells, its expression is a common marker of recent neuronal activity.^[^ ^46^, ^47^, ^48^ ^]^

**Figure 1:**
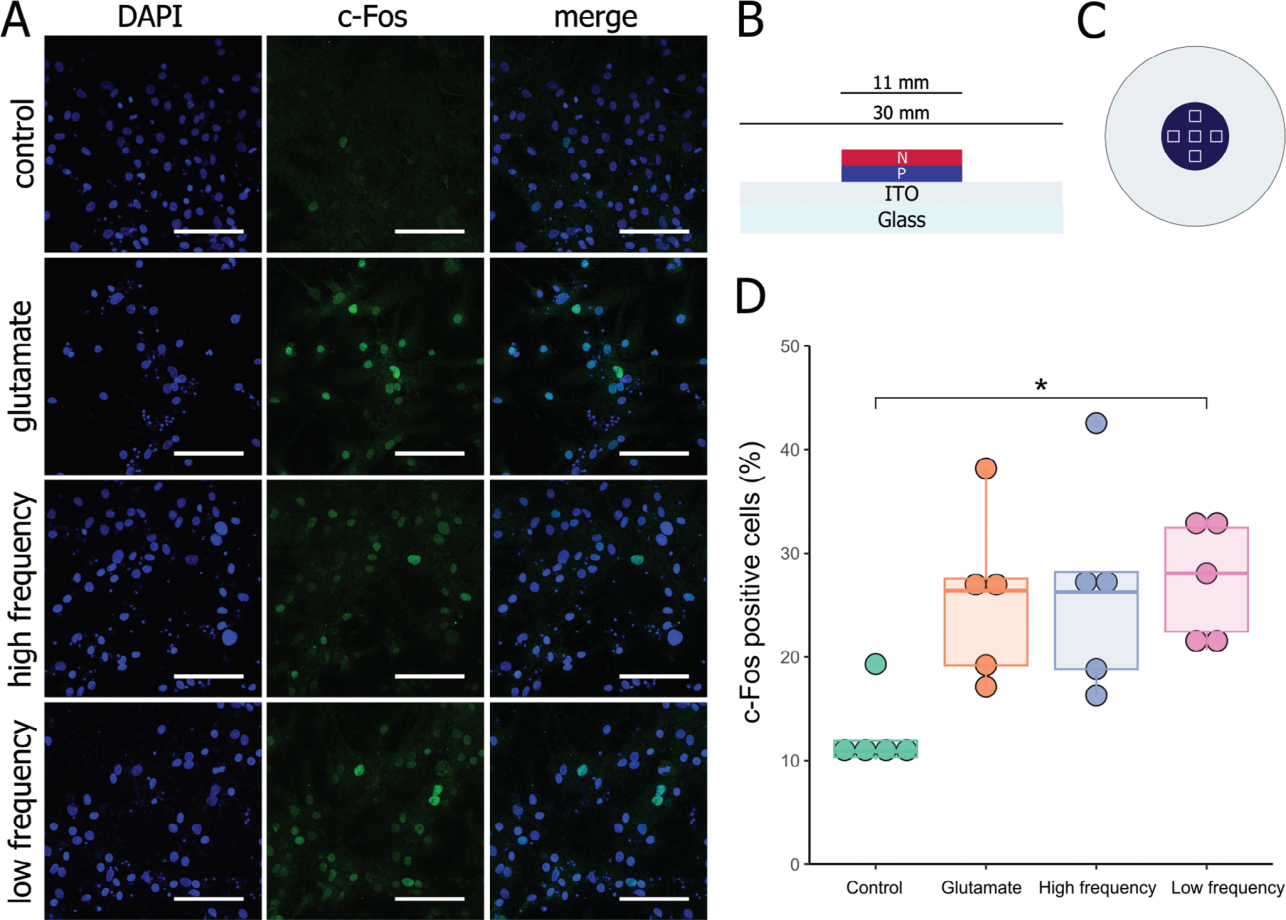
c‐Fos expression in rat primary cortical cells photostimulated with glass‐ITO/OEPC. A) Representative photomicrographs of c‐Fos immunofluorescent staining (green) with DAPI counterstaining of nuclei (blue) following 30 min stimulation protocol or control conditions. “Control” signifies cells left in darkness; “glutamate” – cells left in darkness with the addition of 20 µm L‐glutamic acid; “high frequency” – cells stimulated with red light pulsed at 20 Hz (2 ms pulse); “low frequency” – cells stimulated with red light pulsed at 2 Hz (5 ms pulse). B) Schematic cross‐section of the OEPC used in the experiment. The *p–n* junction serving as a charge‐separating electrode was deposited on an ITO‐covered glass coverslip, functioning as a back electrode. C) Top view of the OEPC. Dark‐blue circle represents the *p–n* layer, whereas light‐silver circle depicts the ITO back electrode. Squares indicate sampling locations for image analysis. D) Percentage of c‐Fos^+^ cells relative to the number of nuclei 60 min following stimulation or control conditions. ^*^ *p* < 0.05. Scale bar: 100 µm.

The effect of light‐controlled electric stimulation on c‐Fos expression in primary cortical cells was first assessed on glass/ITO‐OEPCs following stimulation with low frequency (2 Hz) and high frequency (20 Hz) light pulses (**Figure** **1**A–C). The addition of 20 µm glutamic acid (glutamate) served as a positive control for glutamatergic signaling in the culture. Significant differences in the percentage of c‐Fos‐stained cells were observed amongst the groups (χ^2^(3)  =  9.01, *p*  =  0.03; Figure 1A,D). Although the increase in c‐Fos^+^ cell percentage in both the low and high‐frequency groups was as high as the glutamate group, post hoc comparisons indicated a significant difference only in cells stimulated with low‐frequency light pulses (*p* = 0.03). A potential effect of the light treatment itself was excluded, as the high frequency (20 Hz) stimulation without OEPCs yielded c‐Fos levels comparable to untreated controls. (Figure S3, Supporting Information).

**Figure 2:**
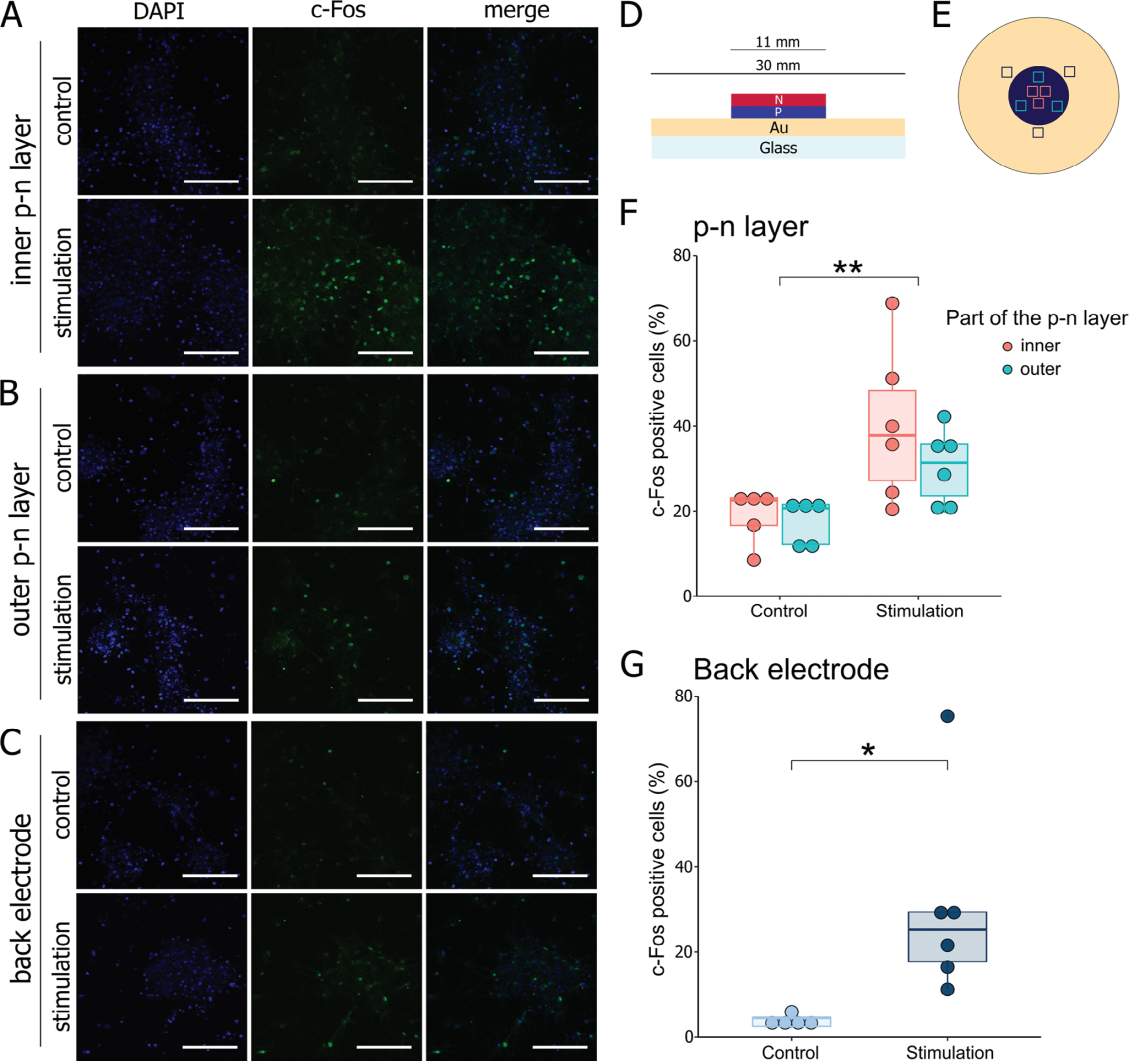
c‐Fos expression in rat primary cortical cells photostimulated with glass‐Au/OEPC. A,B,C) Representative photomicrographs of c‐Fos immunofluorescent staining (green) with DAPI counterstaining of nuclei (blue) following 30 min stimulation protocol or control conditions. “Control” signifies cells left in darkness; “stimulation” – cells stimulated with red light pulsed at 20 Hz (2 ms pulse). A) Photomicrographs of cells located on the inner part of the *p–n* layer. B) Photomicrographs of cells located on the outer part of the *p–n* layer. C) Photomicrographs of cells located on the back electrode. D) Schematic cross‐section of the OEPC used in the experiment. E) Top view of the OEPC. A dark‐blue circle represents the *p–n* layer, whereas gold circle depicts the gold back electrode. Squares indicate sampling locations for image analysis in respect of their location: pastel red – inner part of the *p–n* layer; turquoise – outer part of the *p–n* layer; dark blue – back electrode. F,G) Percentage of c‐Fos^+^ cells relative to the number of nuclei 60 min following stimulation or control conditions. F) Percentage of c‐Fos^+^ cells on the *p–n* layer. G) Percentage of c‐Fos^+^ cells on the back electrode. ^*^ *p* < 0.05, ^**^ *p* < 0.01. Scale bar: 100 µm.

In the first experiment on glass/Au‐OEPC, primary cortical cells were seeded predominantly on the organic *p–n* layer, with a margin of cells reaching the metal back electrode (**Figure** **2**A–E). In this experiment, we aimed to explore the influence of cell localization on top of the photoactive *p–n* layer – whether centrally positioned or situated at the periphery, corresponding to distinct locations within the electric field – on neuronal activity. For this purpose, we conducted a comparative analysis of c‐Fos staining in the inner and outer regions of the *p–n* layer for both stimulated and non‐stimulated groups (Figure 2A,B). Two‐way ANOVA confirmed statistically significant increases of c‐Fos^+^ cells in cultures subjected to light stimulation (Figure 2F; F(1, 18)_Group_  =  12.74, *p* = 0.002) without a detectable difference between the center and periphery of the *p–n* layer (F(1, 18)_Part_  =  1.48, *p* = 0.24). Further, the percentage of c‐Fos^+^ cells on the back electrode (Figure 2C) also significantly increased in the stimulated group as compared to the controls (Figure 2G; W = 2, *p* = 0.017).

**Figure 3:**
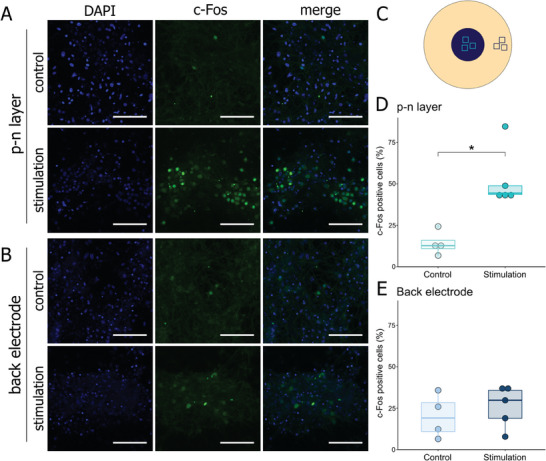
c‐Fos expression in rat primary cortical cells photostimulated with glass‐Au/OEPC with two separated cultures. A,B) Representative photomicrographs of c‐Fos immunofluorescent staining (green) with DAPI counterstaining of nuclei (blue) following 30 min stimulation protocol or control conditions. “Control” signifies cells left in darkness; “stimulation” – cells stimulated with red light pulsed at 20 Hz (2 ms pulse). A) Photomicrographs of cells located on the *p–n* layer. B) Photomicrographs of cells located on the back electrode. C) Top view of the OEPC and schematic representation of the experiment. Two cell clusters were cultured on top of the *p–n* layer and back electrode, physically separated to avoid network signal propagation between the two groups. Squares indicate sampling locations for image analysis in respect of their location: turquoise – *p–n* layer; dark blue – back electrode. D,E) Percentage of c‐Fos^+^ cells relative to the number of nuclei 60 min following stimulation or control conditions. D) Percentage of c‐Fos^+^ cells on the *p–n* layer. E) Percentage of c‐Fos^+^ cells on the back electrode. ^*^ *p* < 0.05. Scale bar: 100 µm.

We further aimed to investigate whether the stimulation of cells out of *p–n* layer was induced by stimulation through the back electrode or due to the signal propagation in neuronal networks from the photoactive layer. For this purpose, we seeded two separate primary cortical cell clusters, one on top of the *p–n* layer and one on the surface of the gold back electrode (**Figure** **3**A–C). Stimulation‐induced c‐Fos increases were detected exclusively in the cell clusters on the p–n layer (Figure 3A,D; *p* = 0.014), ruling out an anodic stimulation through the back electrode (Figure 3B, E; *p* = 0.51).

### 2.3 OEPC Implantation on the Cortical Surface and Tissue Response

**Figure 4:**
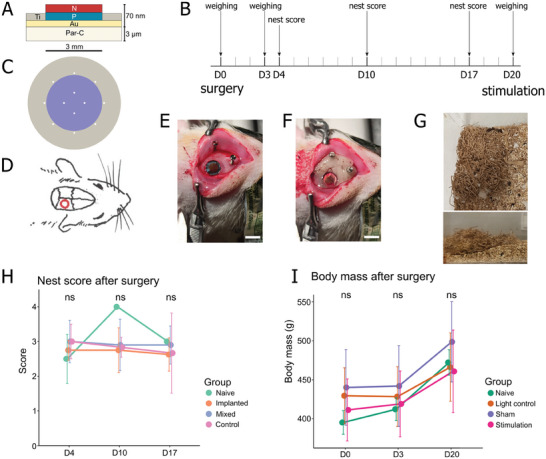
Experimental planning, surgical procedure, and post‐surgery animal welfare during a long‐term OEPC implantation. A) Schematic cross‐section of a flexible OEPC implant used in in vivo experiments. B) Experimental planning for chronic OEPC implantation. Animals underwent light stimulation 3 weeks post‐surgery. Simultaneously, body mass (day [D]0, D3, and D20) and nest scores (D4, D10, D17) were analyzed to assess animal welfare. C) Top view of the flexible OEPC used for implantation. The purple circle represents the *p–n* layer, the grey circle depicts the back electrode, and white dots depict pores (100 µm diameter). D) Schematic of craniectomy and OEPC implantation. E) Intraoperative view of OEPC placed on top of exposed right somatosensory cortex.F) Intraoperative view of OEPC closed with a window (5 mm diameter) made of transparent resin and embedded in dental cement. G) Representative pictures of a nest built by OEPC‐implanted animals on D4, viewed from the top and side of the cage. The pictures depict a nest of good quality (score = 3). H) Comparison of nest scores during a three‐week OEPC implantation. Nest quality was scored in each cage housing two animals: “Implanted” (*n* = 4 cages), “Control” (surgery without OEPC implantation; *n* = 3 cages), “Mixed” (one implanted + one surgery control; *n* = 5 cages), “Naive” (age‐matched males; *n* = 2 cages). No statistically significant difference was observed between the time points or the groups. I) Timeline of body mass change in animals following the surgery. A consistent increase of body mass over time was observed, while no differences between the groups could be discerned. “Light control” – light treatment without OEPC (*n* = 5); “Sham” – no light treatment with OEPC (*n* = 5); “Stimulation” – light treatment with OEPC (*n* = 6), “Naive” – age‐matched males (*n*  =  4). ns – not statistically significant. Scale bar: 5 µm.

Round parylene‐OEPCs (⌀: device 5 mm; *p–n* 3 mm) with pores that allow for cerebrospinal fluid exchange were implanted on the cortical surface after a craniectomy and durectomy. The implantation site of this study (parietal cortex) is relatively flat, however, the parylene‐OEPCs could be implanted on the brain regions characterized by much higher curvature (Figure S4, Supporting Information). The craniectomized hole was closed using a transparent medical‐grade polymer creating a cranial window, which was stabilized by dental cement for acute and semi‐chronic implantations (**Figure** **4**A–F). Surgery and implantation was well tolerated by all groups, substantiated by nest scores comparable to naïve animals (F(3, 10)_Group_ = 1.138; *p* = 0.38, F(2, 20)_Time_ = 1.004; *p* = 0.384; Figure 4G,H) and similar weight gain (F(2, 15)_Group_ = 0.734; *p* = 0.5, Figure 4I) across three weeks post‐surgery.

**Figure 5:**
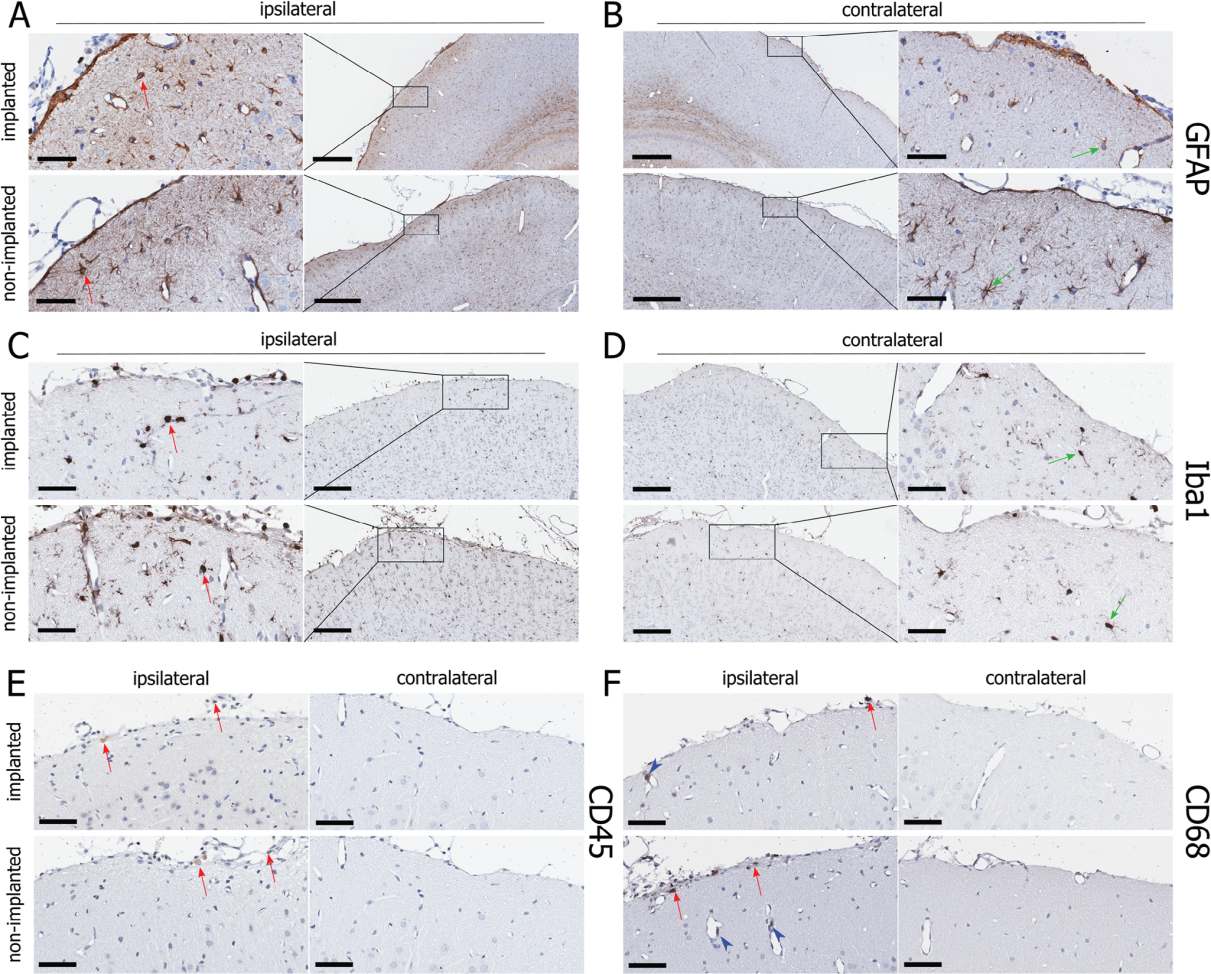
Characterization of foreign body response three week post‐implantation in the somatosensory cortex. A) GFAP immunoreactivity in OEPC‐implanted (upper panel) and non‐implanted animals (lower panel) in the ipsilateral hemisphere. Red arrows – GFAP^+^ cells displaying a reactive morphology. B) GFAP immunoreactivity in OEPC‐implanted (upper panel) and non‐implanted animals (lower panel) in the contralateral hemisphere. Green arrows – GFAP^+^ cells not showing marks of reactivity. C) Iba1 immunoreactivity in OEPC‐implanted (upper panel) and non‐implanted animals (lower panel) in the ipsilateral hemisphere. Red arrows – Iba1^+^ cells displaying a reactive morphology. D) GFAP immunoreactivity in OEPC‐implanted (upper panel) and non‐implanted animals (lower panel) in the contralateral hemisphere. Green arrows – Iba1^+^ cells not showing marks of reactivity. E) CD45 immunoreactivity in OEPC‐implanted (upper panel) and non‐implanted animals (lower panel). In both animal groups, single leukocytes were observed on the surface of the brain, predominantly in the fibrotic tissue close to the surgery site (left panel). The contralateral site remained devoid of CD45^+^ cells (right panel). F) CD68 immunoreactivity in OEPC‐implanted (upper panel) and non‐implanted animals (lower panel). In both animal groups single monocytes/macrophages were observed on the surface of the brain and in the blood vessels in the brain tissue (left panel). The contralateral site remained devoid of CD68^+^ cells (right panel). Scale bar: (A, right panel; B, left panel): 400 µm; (A, C – left panel; B, D – right panel): 50 µm; remaining pictures: 200 µm.

Immunohistochemical analyses of glial and immune cell markers three weeks after the surgery and OEPC implantation showed a high tolerance toward the device. We observed more pronounced staining of an astrocyte marker (GFAP) and a higher number of GFAP^+^ cells in the ipsilateral somatosensory cortex, particularly in the most superficial cortical layer near the surgical site, compared to the contralateral side (**Figure** **5**A,B). GFAP^+^ cells in the ipsilateral side showed morphological features of reactive astrogliosis^[^ ^49^, ^50^ ^]^ with a more intense GFAP‐staining and slight cell body hypertrophy (Figure 5A, left panel, red arrows), whilst those in contralateral hemisphere were typical astrocytes (Figure 5B, right panel, green arrows). Microglial staining (Iba1) exhibited a similar pattern. Iba1^+^ cells showed a reactive microglial morphology with a rounder shape, fewer processes, and lower ramification in the ipsilateral cortex (Figure 5C, left panel, red arrows) compared to the resident microglia observed in the contralateral side (Figure 5D, right panel, green arrows). Leukocytes (CD45^+^) and specifically macrophages (CD68^+^) were exclusively observed in the ipsilateral cortex, weak in staining, sparse in number, and mostly localized in the fibrous tissue covering the surface near the surgical site (Figure 5E,F, red arrows). Although few macrophages were detected inside the walls of cerebral blood vessels (Figure 5F, blue arrowheads), neither CD45^+^ nor CD68^+^ cells were detected in the brain parenchyma. OEPC‐implanted animals did not show a detectable difference compared to the controls with regard to any of these inflammatory markers, suggesting that observed inflammatory responses were in large attributable to the surgical procedure, but not the implantation.

### 2.4 Neuronal In Vivo Activation by OEPC Stimulation

**Figure 6:**
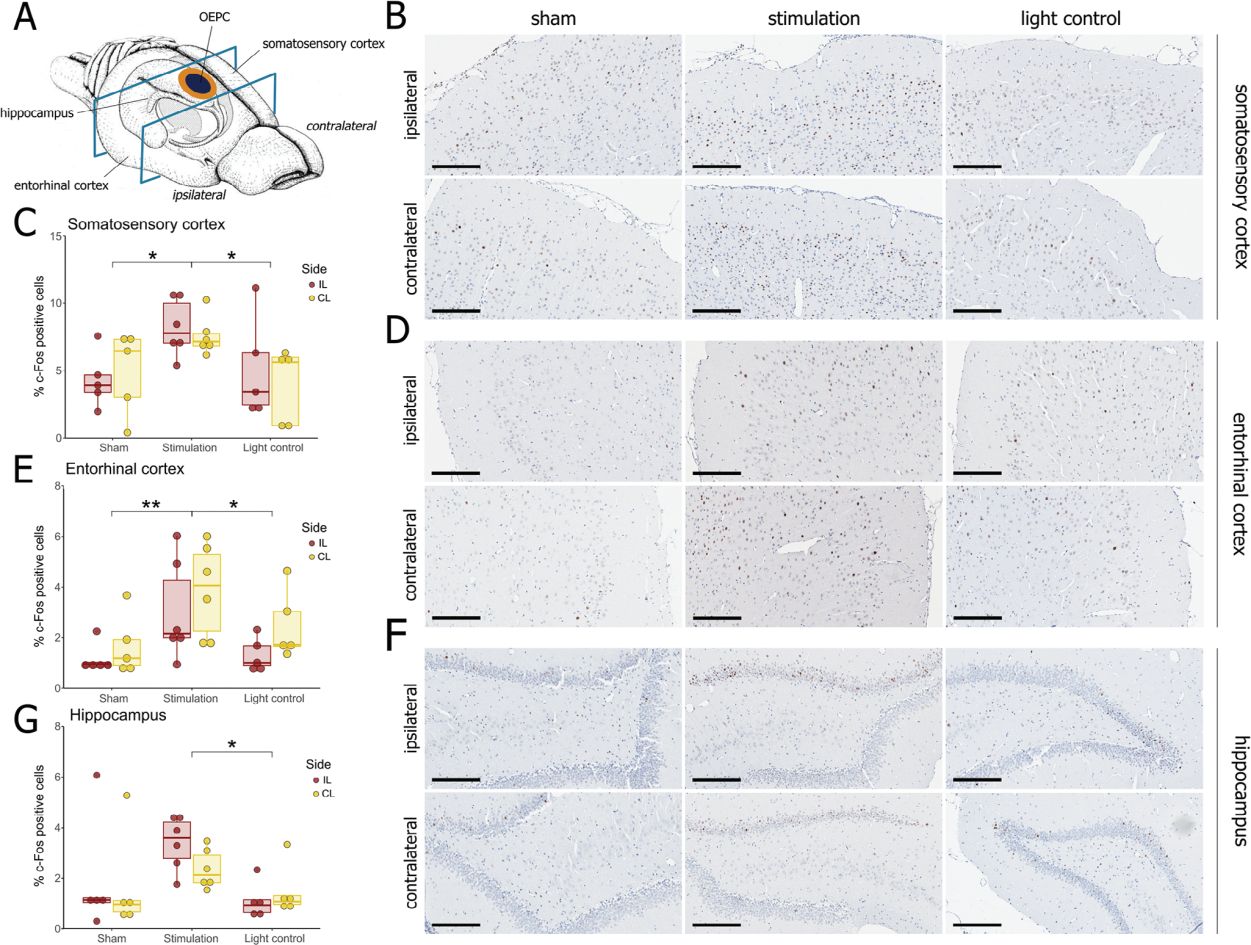
c‐Fos expression in animals subjected to light treatment (638 nm laser; 20 Hz, 2 ms pulse; 30 min) following long‐term OEPC implantation. A) Graphical illustration of OEPC placement (orange‐dark blue circle) and section sampling part (between the blue rectangles). Images were sampled in the somatosensory cortex, entorhinal cortex, and hippocampus in both ipsi‐ and contralateral hemispheres. Reproduced under terms of the CC‐BY license.^[^ ^51^ ^]^ 2024, Cheung and Cardinal, published by BMC.^[^ ^52^ ^]^ B) Representative photomicrographs of DAB immunostaining of c‐Fos in the somatosensory cortex. Discernible c‐Fos^+^ cells in the layers II‐IV. C) Percentage of c‐Fos^+^ cells in the somatosensory cortex. D) Representative photomicrographs of DAB immunostaining of c‐Fos in the entorhinal cortex. Discernible c‐Fos^+^ cells throughout all the layers. E) Percentage of c‐Fos^+^ cells in the entorhinal cortex. F) Representative photomicrographs of DAB immunostaining of c‐Fos in the dentate gyrus of the hippocampus.Discernible c‐Fos^+^ cells in the upper blade of the dentate gyrus. G) Percentage of c‐Fos^+^ cells in the hippocampus. “Light control” – animals without OEPC subjected to light treatment; “Sham” – animals with implanted OEPC subjected to sham treatment; “Stimulation” – animals with implanted OEPC subjected to light treatment. ^*^ *p* < 0.05. Scale bar: 200 µm.

Due to potentially confounding factors associated with the surgery itself, such as pain signaling, wound healing, and inflammation, quantification of c‐Fos expression shortly after OEPC implantation proved challenging (Figures S5–S8, Supporting Information). In the semi‐chronic group, where the healing process was already finished, we assessed c‐Fos expression in three regions (somatosensory cortex, entorhinal cortex, and hippocampus) in the ipsi‐ and contralateral hemispheres (**Figure** **6**A).

In the somatosensory cortex, the percentage of positive cells was significantly higher in the light‐stimulated, OEPC‐implanted animals (Figure 6B, middle panel) compared to the sham‐treated, OEPC‐implanted rats (Figure 6B, left panel; *p* = 0.018) and to the animals subjected to the light treatment without OEPC implantation (Figure 6B, right panel; *p* = 0.015). Statistical analysis of positive cells revealed a significant effect of treatment on c‐Fos expression (Figure 6C; F(2, 26)_Group_  =  6.099, *p* = 0.0067). No significant differences were observed between the ipsi‐ and contralateral sides (F(1, 26)_Side_  =  0.184, *p* = 0.67).

In the entorhinal cortex, an increase of c‐Fos^+^ cells in the stimulated group (Figure 6D, middle panel) was evident compared to the sham (Figure 6D, left panel; *p* = 0.0072) and to the light controls (Figure 6D, right panel; *p* = 0.043). The effect of the OEPC stimulation was statistically significant (Figure 6E; F(2, 26)_Group_ = 6.2; *p* = 0.0063). Ipsi‐ and contralateral sides showed comparable c‐Fos^+^ cell populations (F(1, 26)_Side_  =  2.77, *p* = 0.11), though cell counts at the contralateral side were slightly higher in all treatment groups.

In the hippocampus, a c‐Fos expression was increased in the stimulation (Figure 6F, middle panel) compared to the light control (Figure 6F, right panel; *p* = 0.04) groups. Differences in c‐Fos^+^ cell percentage observed between the groups were statistically significant (Figure 6G; F(2, 26)_Group_ = 3.474, *p* = 0.046), A difference was also discernible between the stimulation and sham groups, although it was statistically not significant (Figure 6F, left panel; *p* = 0.22). Between the two sides of the brain, no significant difference was observed (F(1, 26)_Side_ = 0.477, *p* = 0.5), though we were able to discern a slight trend to higher percentages of c‐Fos^+^ cells in the ipsilateral hemisphere.

### 2.5 Characterization of Cells Expressing c‐Fos Following OEPC Stimulation

In the somatosensory cortex, an examination of immunostained brain sections from the animals in the stimulated group unveiled the highest abundance of c‐Fos^+^ cells in the superficial cortical layers, spanning approximately from layer I to layer IV, with the majority of cells located in layer II/III (Figure 6B). In the entorhinal cortex, c‐Fos^+^ cells were observed throughout all cortical layers (Figure 6D), especially in the contralateral hemisphere. Within the hippocampal regions, c‐Fos expression was most prominent in the dentate gyrus (DG), particularly in its upper (suprapyramidal) blade, and to a lesser extent in the hilus of the DG and in Cornu Ammonis (CA) 3 (Figure 6F). Only single positive cells were observed in CA1 (Figure S9, Supporting Information).

**Figure 7:**
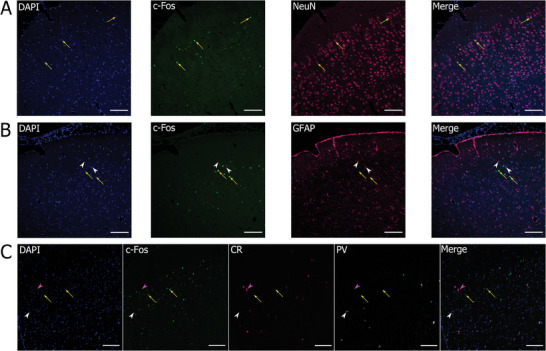
Characterization of c‐Fos^+^ cells in the ipsilateral somatosensory cortex of OEPC‐stimulated animals. A) Double immunofluorescent staining of c‐Fos (green) and NeuN, marker of differentiated neurons (magenta) with additional DAPI counterstaining of nuclei (blue) revealed co‐expression of the two proteins (arrows), indicative of neuronal expression of c‐Fos. B) Double immunofluorescent staining of c‐Fos (green) and GFAP, a marker of astrocytes (magenta) with additional DAPI counterstaining of nuclei (blue) revealed no co‐expression of c‐Fos (arrows) and GFAP (white arrowheads), with an exception of one animal (shown in Figure S10, Supporting Information). C) Triple immunofluorescent staining of c‐Fos (green) and two common marker of interneurons: calretinin (CR; magenta) and parvalbumin (PV; white) with additional DAPI counterstaining of nuclei (blue). No co‐expression of c‐Fos (arrows) and CR (purple arrowheads) or PV (white arrowheads) was observed. Scale bar: 100 µm.

To unravel specific brain cell types expressing c‐Fos, we performed multiple IF staining of the protein with common neuronal and glial markers. Across all sections from the selected animals, c‐Fos co‐expressed with NeuN in both the ipsi‐ and contralateral somatosensory cortex and in the hippocampus, consistent with its characterization as a marker of neuronal activity (**Figure** **7**A). c‐Fos^+^‐GFAP cells were not observed in any of the investigated regions (*n* = 8 animals, Figure 7B), except in one animal, where few cells were present near the site of contact with the parylene‐OEPC implant (Figure S10, Supporting Information). Similarly, calretinin^+^ or parvalbumin^+^ interneurons did not show c‐Fos expression in the investigated regions (Figure 7C). Taken together these findings suggest that, based on the shape and localization of the c‐Fos^+^ cells in the brain tissue, it can be inferred, that the main cell type responsive to the light‐controlled electric stimulation via OEPCs at given parameters is a major excitatory projection neuron, particularly a layer II/III pyramidal cell, which renders the applied stimulation largely excitatory.

### 2.6 OEPC Durability After the Explantation

**Figure 8:**
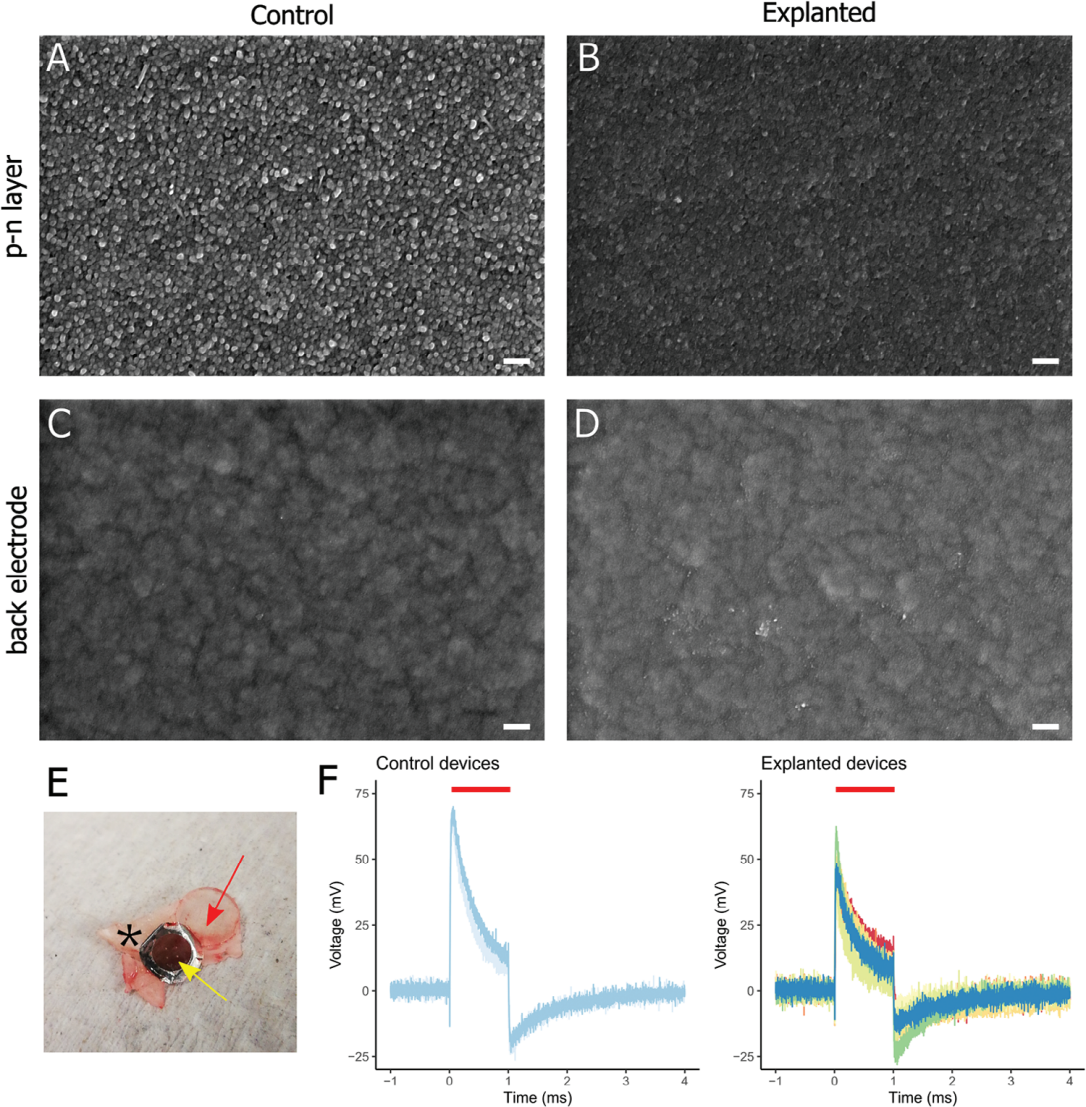
Characterization of flexible OEPC explanted three weeks post‐surgery. A–D) Representative SEM photomicrographs of the surface of the *p–n* layer A,B) and back electrode C,D) of the devices removed directly from the wafer A,B) and explanted from the animals B,D). E) Representative macroscopic photograph of an explanted OEPC (yellow arrow) with a transparent resin window (red arrow) and a fragment of dental cement (asterisk). F) Measurement of transient voltage of control (left) and explanted (right) devices. Visible charging during illumination (red bar) with consecutive discharging phase remain clearly discernible in both groups. Control devices, *n* = 2; explanted devices *n* = 9. Scale bar: 200 nm.

Following parylene‐OEPC explantation, we assessed device survival in terms of its material composition and functionality. Material stability was examined at the microscopic level through SEM imaging (**Figure** **8**A–D). We visually inspected the surfaces of the photoactive *p–n* layer and the Au/Ti back electrode in devices obtained from animals three weeks after implantation, as well as in control devices freshly removed from the wafer. The *p–n* layer consists of tightly‐packed columnular nanocrystal domains, giving a distinctive morphology^[^ ^27^ ^]^. In comparison to the control devices (Figure 8A), we observed a smoother surface of the *p–n* layer in the explanted parylene‐OEPC (Figure 8B). Nevertheless, the nanocrystal domains of the material were still discernible in the explanted devices. No signs of delamination, cracking, or pores were observed. The *p–n* surface did not show additional changes in the close vicinity of attached tissue (Figure S11, Supporting Information) compared to parts of the layer devoid of tissue debris. No apparent differences in surface appearance were observed between the explanted and control parylene‐OEPC (Figure 8C,D). Overall, our observations suggest changes indicative of minor mechanical abrasion or adhesion of proteins and organic compounds, which form a layer on top of the *p–n* accounting for a smoother morphology.

A functional analysis of the explanted devices was carried out by measuring voltage time courses. Only macroscopically intact devices were designated for further characterization (Figure 8E), i.e. those that were not broken during explantation. The transient voltages evoked by 660 nm light pulses exhibited only a minor decrease in the initial maximum voltage peak of the parylene‐OEPC implant after three weeks in animals as compared to the control devices freshly removed from the wafer (Figure 8F). Both groups of devices displayed a typical charging/discharging curve with a distinct peak photovoltage, indicating high stability of the parylene‐OEPC within the animal body. The small decrease in photovoltage may be caused by the surface morphology alterations visible from the SEM imaging.

### 2.7 Temperature Measurements Following Light Stimulation

To better understand temperature changes during the light stimulation in vivo, we assessed the heat distribution using infrared thermography in a brain phantom (Figure S12A, Supporting Information). Laser stimulation did not cause any notable increase in temperature in the simulated tissue near the parylene‐OEPC (Figure S12B, Supporting Information; black dashed circle, representative point measurement: 32.7 °C) in comparison to the surrounding brain phantom (Figure S12B, Supporting Information; blue dashed circle, representative point measurement: 32.9 °C). Compared to the unstimulated control measurement, the stimulated phantom showed an increase of the average surface temperature of Δ*T* = 0.1 °C.

## 3 Discussion

The OEPC is a wireless platform for neurostimulation that has recently been validated for chronic stimulation of a peripheral nerve target.^[^ ^27^ ^]^ This encouraged us to engineer a version of the OEPC suitable for chronic cortical implantation and wireless stimulation. Prior to in vivo implantation, we performed a series of experiments to test the efficacy and safety of stimulation using neuronal cell cultures.

### 3.1 Neuronal Activation Following OEPC Stimulation in Cell Culture

Visual inspection and LDH cytotoxicity test of the rat primary cortical cell culture showed excellent survival of cells cultured on the surface of OEPCs, similar to the control culture on glass coverslips (Figure S2, Supporting Information). No cytotoxic effect of OEPC in the cortical culture was found over a cultivation period of 14 days. This is in line with our previous studies,^[^ ^33^, ^35^ ^]^ where hippocampal neurons or chicken retinas were cultivated on OEPC surfaces with similar composition. Observed alterations in LDH activity over time correspond well to the normal growth and maturation of cortical cultures, including increases in LDH activity at DIV10 reflecting the delayed death of proliferating cells by prior mitosis inhibition. However, it is worth to note that a significant reduction was observed in the LDH activity after stimulation at DIV14 in comparison to that prior to the stimulation. This effect could not be attributed to the natural decay of LDH as it was observed exclusively in the stimulated group. Furthermore, LDH has an ≈9 h half‐life in culture medium,^[^ ^53^ ^]^ which significantly surpasses the experimental time frame here (90 min from the onset of light treatment to sample collection). Another mechanism contributing to a rapid reduction of the LDH levels might be its inactivation by chemical compounds present in the medium. Hydrogen peroxide and reactive oxygen species are known to be generated upon illumination of photoactive organic semiconductors in simple buffers^[^ ^34^, ^54^, ^55^, ^56^, ^57^ ^]^ and their inactivating effect on dehydrogenases has been previously documented.^[^ ^58^, ^59^ ^]^ Nevertheless, whether reactive oxygen species are generated during stimulation in cell culture medium necessitates further exploration.

OEPC stimulation has been previously investigated in terms of changes in membrane voltage in cells cultivated on their surfaces,^[^ ^33^, ^34^ ^]^ generation of action potentials in neurons,^[^ ^35^ ^]^ and activity synchronization in cardiac cells.^[^ ^60^ ^]^ However, most of these studies have predominantly focused on the effects of the stimulation in the millisecond range at the single‐cell level. Here we set up an experimental design that allows detection of possible temporal summation of subthreshold depolarization, and adds to our understanding of OEPCs functionality in a neuronal network after a period of stimulation, substantiated by c‐Fos expression.

In our initial experiment, neurons were subjected to two stimulation protocols: low‐frequency light with a longer pulse, as previously described in our patch‐clamp study,^[^ ^35^ ^]^ and high‐frequency light with a shorter pulse, intended for use in our in vivo study (Figure 1). The c‐Fos expression in cell culture treated with both stimulation protocols reached similar levels as in cells treated with pure neurotransmitter (L‐glutamic acid), serving as positive control. Although low‐frequency stimulation yielded a slightly higher increase in percentage of c‐Fos‐expressing cells, we opted for a high‐frequency protocol, to constrain potential activation of inhibitory cells,^[^ ^61^, ^62^, ^63^ ^]^ which could have interfered with our in vivo experimental design.

Subsequent experiments on cortical cell culture aimed to investigate the impact of the localization of the culture on the device with respect to neuronal activation. Culture seeded on the entire *p–n* layer, with a margin of cells cultivated on the surface of the back electrode, showed an increase of activity measured in terms of c‐Fos expression on both parts of the device (Figure 2). Neuronal activity on the *p–n* layer was independent of the cells’ position on the layer. However, when two separate clusters of cells were cultivated – one on top of the *p–n* layer, one on the back electrode – only the cluster on the photoactive *p–n* layer showed an increase in c‐Fos^+^ cells (Figure 3). This observation suggests that activation of cells on the back electrode in the first experiment could arise from the signal propagation from the neurons located on the *p–n* layer through vast connections, and the electrical stimulation of the back electrode itself is not strong enough to trigger neuronal activity. Therefore, it is reasonable to infer that the OEPC primarily functions through rapid cathodic charging of the *p–n* layer, with any potential stimulation of the metal back electrode having a very constrained effect.

### 3.2 Neuronal Activation Following OEPC Stimulation in the Animal Model

In the brain tissue, OEPC stimulation induced widespread activation of neurons in all examined regions (Figure 6). Notably, there were no discernible differences between the ipsilateral cortex and contralateral cortex, implying the putative signal propagation over callosal connections (Figure 6B,C). The widespread activation observed in the contralateral somatosensory cortex aligns with expectations, as cortical cells establish both homotopic (within the same cortical layer) and heterotopic (extending to another layer) connections with their contralateral counterparts.^[^ ^64^, ^65^ ^]^ The depth of the voltage change within the tissue seems sufficient to reach layers II/III of the somatosensory cortex, where the majority of neurons projecting through the corpus callosum are located.^[^ ^66^ ^]^

We also observed an increased c‐Fos expression in two regions of the parahippocampal formation: the entorhinal cortex (Figure 6D,E) and the hippocampus (Figure 6F,G). The mechanism of hippocampal activation can be attributed either to direct stimulation through the OEPC or to indirect activation via neuronal connections extending from the somatosensory cortex. While it may be technically feasible to directly stimulate regions beneath the cortex, our observations do not fully substantiate this possibility. We seldom detected c‐Fos expression in the deeper layers (V/VI) of the somatosensory cortex, and only isolated c‐Fos^+^ cells were observed in the most dorsal portions of the hippocampus, indicating a more superficial effect of the OEPC stimulation. Although we did not model the electric field generation for the devices in this study, a separate model of OEPC stimulation in mouse cortex failed to predict voltage changes in subcortical brain regions, reaching a maximal potential of about 0.15 V in deep cortical regions, but not crossing the corpus callosum.^[^ ^36^ ^]^

An alternative explanation for the increased c‐Fos expression in the hippocampus is signal propagation within the neuronal networks. The hippocampus receives somatosensory input from the entorhinal cortex to the gyrus of the DG,^[^ ^67^, ^68^ ^]^ and the signal is further propagated to CA3 and then to CA1 through the trisynaptic circuit characteristic of this brain region. From CA1, the signal returns to the entorhinal cortex, specifically its layer V, completing the corticohippocampal loop.^[^ ^69^ ^]^ In the hippocampus, the highest number of c‐Fos expressing cells was noted in the DG, particularly in its upper blade. Fewer cells were detected in the CA3 and the CA1. This pattern suggests a robust signal reaching the initial segment of the hippocampal circuit, gradually diminishing along the loop. However, c‐Fos immunoreactivity was observed in all layers of the entorhinal cortex, indicating neuronal activity in the entire corticohippocampal loop. Observed lower c‐Fos expression in CA reflects the intrinsic hippocampal inhibitory mechanism via inhibitory interneurons located at the CA3 and CA1 regions.^[^ ^70^, ^71^ ^]^

We observed an indirect activation of the connected deeper brain regions and contralateral hemisphere through OEPC stimulation. It should be noted that the size of the OEPC used in this study was large enough to be considered a bulky electrode at the cortical surface with respect to the small size of the rat brain, leading to an extensive secondary stimulation due to increased network activity. Such secondary stimulation is desirable to activate sites/nerves that are remote or inaccessible without an invasive intervention at the site of interest. For instance, the cortical stimulation in the contralateral hemisphere holds potential advantages for the treatment of trauma or stroke, with the implant positioned on the healthy hemisphere and the evoked signal propagating to the affected region. This approach has been already tested in an optogenetic mouse model of stroke, where stimulated animals showed a significantly faster recovery phase, with increased expression of protective neurotrophins, markers of plasticity, and significantly better performance in sensory‐motor behavior tests.^[^ ^72^ ^]^ Contralesional theta‐burst stimulation following stroke also led to a faster and better recovery of the upper limb mobility in human patients.^[^ ^73^ ^]^ Whether similar benefits are achievable using OEPC remains to be explored. Moreover, it is crucial to consider potential off‐target effects, which could be involved during the stimulation. The flexibility in design and production of these devices allows for the targeting of specific neuronal populations in cortical regions of interest to encode specific stimulation information, in case a strong or widespread secondary stimulation is not desired. Since the created electric field depends on size and shape of the *p–n* layer, as well as the light intensity used for stimulation, it is plausible to anticipate that the extent of the secondary stimulation could be tuned by manipulating the primary stimulation, although this point was not addressed in our study.

Further we characterized the type of cells expressing c‐Fos with multiple immunostainings of common neuronal and astrocytic markers in brain sections after stimulation (Figure 7). As anticipated, c‐Fos was present almost exclusively in neuronal cells (Figure 7A,B). This observation reinforces the claim that the increase in c‐Fos expression is a result of the OEPC stimulation, and rules out a confounding effect of a possible surgery‐induced inflammatory proliferation of astrocytes, by which an astrocytic c‐Fos expression is known to be induced.^[^ ^74^ ^]^ The process of astrocytic proliferation and/or differentiation, however, may provide an explanation for the circumscribed c‐Fos expression in astroglial cells we observed in one animal (Figure S10, Supporting Information).^[^ ^74^ ^]^ Additionally, it may also be attributed to glutamatergic activation^[^ ^75^ ^]^ or inflammatory signaling.^[^ ^76^ ^]^

We did not observe c‐Fos co‐expression with markers of interneurons: parvalbumin and calretinin (Figure 7C). Parvalbumin is predominantly expressed in basket cells, located in the layer IV of neocortex and targeting soma and proximal dendrites of pyramidal cells, and in chandelier cells, connecting mainly to the initial axon segment of pyramidal cells.^[^ ^77^ ^]^ Calretinin marks mostly interneurons located in the cortical layers II/III that interact with distal dendrites of pyramidal cells.^[^ ^77^ ^]^ Selective activation of excitatory cells may stem from stimulation of their processes that extend toward the surface, whereas the investigated types of interneurons possess shorter, localized processes.^[^ ^78^, ^79^ ^]^ Selective stimulation of excitatory cells through cortical stimulation of the processes was predicted by mathematical models^[^ ^80^, ^81^ ^]^ and our observations seem to substantiate these predictions. Additional process explaining lack of activation might be prolonged afterhyperpolarization of interneurons in the cortex, which was described to be induced by electrical stimulation.^[^ ^82^, ^83^, ^84^ ^]^

### 3.3 Foreign Body Response and Stability of OEPC Following Semi‐Chronic Implantation

In our previous investigation focusing on chronic OEPC implantation in the sciatic nerve, we observed a sustained device functionality with no detrimental histological changes over 100 days post‐surgery.^[^ ^27^ ^]^ Nevertheless, the intricacies of brain tissue necessitated a thorough examination of both tissue response to the implant and stability of the device following the implantation.

Immunostaining with common glial and immune cell markers revealed discernible differences in cell number and morphology between the ipsilateral and contralateral sides (Figure 5). We observed broader and more intense immunoreactivity of astrocyte (GFAP) and microglia (Iba1) markers near the surgical site, particularly in the superficial layers of the cortex (Figure 5A–D), which indicated increased proliferation and migration of those cell types. Moreover, astrocytes and microglia present in this region exhibited morphological hallmarks of reactivity, such as round shape, cell body hypertrophy, and disappearance of fine distal processes.^[^ ^49^, ^50^ ^]^ Importantly, these characteristics were consistently observed in both animals implanted with OEPC and in control rats that underwent craniectomy and durectomy, but were left without an implant. Therefore, it is reasonable to conclude that the cellular response was initiated by the surgical procedure itself, and the presence of the implant did not cause a significant, moderate to strong foreign body response. The astrocytic activation was not sufficient to result in the glial scar formation, a process typically completed in the central nervous system within 14 days post‐injury.^[^ ^85^ ^]^ The absence of a significant glial scar formation is crucial, as it could substantially impede electric field distribution in the brain tissue.

Similarly, immunostaining for markers of non‐brain native immune cells (panleukocytic marker CD45 and monocyte and macrophage marker CD 68) did not exhibit differences in the cell numbers between the implanted and non‐implanted animals (Figure 5E,F). Immunoreactive cells were exclusively present in the ipsilateral cortex, proximal to the injury site – predominantly on the brain surface, with isolated cells visible in the walls or the lumen of the blood vessels. Notably, the brain parenchyma itself remained devoid of infiltrating immune cells, typically associated with recruitment during inflammation.^[^ ^86^ ^]^ Hence, it is reasonable to infer that the presence of OEPC did not induce a significant foreign body response, affirming the safety of the devices for semi‐chronic implantation.

Even though lack of moderate or strong foreign body response speaks in favor of the future application of OEPC as implantable stimulation devices, further studies are needed for capturing more subtle differences between implanted and non‐implanted animals, preferably with larger cohort sizes. Moreover, conclusive assessment of OEPC suitability as a chronic implant would require much longer implantation periods, including the time points, by which the device stability could get impaired through degeneration, delamination etc., whereby a response different than that of intact and functional devices might be anticipated.

Another crucial aspect of chronic OEPC implantation was an assessment of device functionality after the three‐week period. The comparison of the explanted devices with those freshly removed from the wafer by the means of SEM revealed smoother surface of the *p–n* layer and back electrode (Figure 8A–D), which can be attributed either to a wear‐off caused by micromovements against the tissue or by adhesion of organic material to the superficial parts of the *p–n* layer. The maintained uniformity and granularity of the surface suggested the lack of significant degradation of the photoactive layer. Additionally, voltage measurements of the explanted OEPC indicated sustained functionality, with the output comparable to that of non‐implanted devices (Figure 8F).

The assessment of the thermal effects of the laser operation indicated that OEPC stimulation in this configuration should not pose a risk to adjacent brain tissue. This finding is crucial for the safe application of optoelectronic implants in neural stimulation therapies and aligns with control experiments from our previous studies.^[^ ^27^, ^35^ ^]^ The use of Phytagel brain and 3D‐printed skull phantoms provides a reliable and ethical alternative to animal testing, enabling accurate thermal assessments without the need for live subjects.

Since the perfusion of the brain was not replicated in this brain phantom model, the absence of a measurable heat rise in this setup suggests that any thermal effects could be even less significant in an in vivo setting. The lower surface temperature in close proximity to the OEPC can be attributed to the tolerance or deviation of the measurement setup. It is important to note that this was a single measurement; for more reliable results and a more conclusive statement, additional experiments should be conducted. However, the presented data serve as a promising preliminary indication that laser‐stimulated OEPC may be safely used in neural stimulation therapies, warranting further investigation under controlled and replicated experimental conditions.

In summary, we observed retained device functionality, tissue conformity as well as operational safety. Nevertheless, it is clear that more studies involving longer implantations are necessary to gain a better understanding of the chronic effects on both the tissue and the devices.

## 4 Conclusion

The OEPC has recently demonstrated its reliability as a light‐controlled, wireless stimulation device, eliciting abundant and widely propagated neuronal activity both in in vitro and in vivo models. In our study, OEPC were applied for the first time for semi‐chronic cortical stimulation, and assessed in their functionality and biocompatibility. The resulting neuronal activation induced molecular changes at the nuclear level, as indicated by increased c‐Fos expression after OEPC stimulation. In the cell culture, we observed cathodic stimulation initiated on the photoactive layer coupled with signal propagation within the neuronal networks.

In the rat model, OEPC stimulation resulted in a substantial increase of c‐Fos expression not only in the immediate vicinity of the implanted device but also in deeper brain regions such as the hippocampal formation and the contralateral hemisphere. The potential for signal propagation from the stimulated site to other brain areas holds promise for treatment protocols targeting cortical or hippocampal injuries from the level of a healthy cortex. However, it remains to be explored, whether such stimulation could be performed in a controlled manner.

Importantly, OEPC implantation did not have a detrimental effect on either the brain tissue or device functionality. These promising results represent a significant milestone in the research of photoactive organic materials as a method for neurostimulation and warrant continued investigation of OEPC in animal and human studies, along with additional characterization of its neuromodulatory potential.

## 5 Experimental Section

Full list of used materials and devices is available in Table S1 (Supporting Information).

### Fabrication of Glass OEPC

The fabrication procedure is previously described in detail in Rand et al.^[^ ^33^ ^]^ and Jakešová et al.^[^ ^34^ ^]^ Briefly, OEPC devices were fabricated by physical vapor deposition of organic pigments H_2_Pc (metal‐free phthalocyanine) and PTCDI (*N,N′*‐dimethyl‐3,4,9,10‐perylenetetracarboxylic diimide) on previously silanized gold or ITO covered glass substrates. H_2_Pc (Alfa Aesar) and PTCDI (BASF) were purified by threefold temperature gradient sublimation in a vacuum of < 1 × 10^−3^ Torr.

OEPC devices were fabricated using physical vapor deposition on chromium/gold or ITO‐coated glass coverslips (⌀: 30mm, thickness 0.4 mm) with the organic region defined by a stainless steel shadow mask. The thickness was controlled in situ using a quartz crystal microbalance. Gold‐ or ITO‐coated substrates were treated with O_2_ plasma (50–100 W; 5–10 min) and immediately placed into a chamber held at 90 °C containing vapor of n‐octyltriethoxysilane (OTS) for 2 h, followed by rinsing with acetone and water, and sonicated in acetone for 15 min to remove multilayers and excess silanization physisorbed on the substrate. The OTS layer was found to improve the adhesion of the organic semiconductor layer, prevent delamination, and produce reliably higher photovoltage than bare substrate. Following rinsing and drying under a nitrogen stream, the samples were placed with appropriate shadow masking (⌀: 11 mm) in an organic materials evaporator (HHV Auto306) for vapor deposition. The pigment layers were deposited at a rate of 1.5 Å s^−1^, first for the *p*‐type layer (H_2_Pc), then for the *n*‐type (PTCDI) layer at a base pressure < 2 × 10^−6^ mbar, to reach a total thickness of 60 nm (30 nm, each *p*‐ and *n*‐type).

### Fabrication of Flexible OEPC

For the purpose of in vivo stimulation, flexible OEPC devices were manufactured. Glass wafers (Siegert wafer, 500 ± 20 µm) were coated with a base layer of parylene‐C (2.5 µm; SCS) grown by chemical vapor deposition (SCS Labcoter PDS 2010). Then, a stack of Pd (1 nm), Au (9 nm), and Ti (30 nm) were deposited via magnetron sputtering (Bestec GmbH). The first photolithography step defined the device outline (all covered with the bottom electrode) and pores (⌀: 100 µm; 4 on photoactive pixel, 8 on counter electrode) for better extracellular liquid exchange. AZ 1518 photoresist spin–coated at 1000 rpm was exposed through a soda lime mask using a SÜSS MicroTec MA8 mask aligner equipped with an i‐line filter. The resist was developed in AZ 400K developer diluted 1:4 in deionized water (diH_2_O). Plasma descum was performed in O_2_ plasma (Diener NANO Plasma Cleaner). The metal layers were etched in a KI/I_2_ (Au, Pd) and HF/H_2_O_2_/H_2_O (Ti) etch mixtures. The parylene‐C layer was etched using reactive ion etching (RIE, Oxford Instruments PlasmaPro 80, 200 W, 50 sccm O_2_, 100 mTorr). Residual resist was stripped in acetone. The p–n organic pixel was patterned by parylene peel‐off technique. an anti‐adhesive layer of Micro 90 soap (2%; International Products) was spin–coated at 1000 rpm and left to air dry before deposition of the sacrificial parylene‐C layer (2 µm). The *p–n* organic pixel area was opened in the next photolithography step. AZ 1518 photoresist spin‐coated at 1000 rpm was used as an etch mask for removing the sacrificial parylene‐C layer by RIE (200 W, 50 sccm O_2_, 100 mTorr). Next, the Ti layer serving the role of a RIE etch stop was removed in HF/H_2_O_2_/H_2_O mixture thus exposing semi‐transparent Pd/Au layer acting as the OEPC bottom contact. The organic *p–n* layer was deposited through the parylene‐C mask. Layers of H_2_Pc (30 nm) and PTCDI (30 nm) were thermally evaporated from resistively heated crucibles (Edwards 306, < 2 × 10^−6^ Torr, rates of 1–6 Å s^−1^,). Finally, the parylene‐C sacrificial layer was gently peeled off under diH_2_O. The wafer was washed in diH_2_O to remove the soap residue. The final device was a circular OEPC (⌀: 5 mm) with Pd/Au/Ti back electrode and a central photoactive *p–n* pixel on Pd/Au (⌀: 3 mm).

### Primary Cortical Cell Culture

Postnatal (P0‐P1) Sprague Dawley rats were cryo‐anesthetized and euthanized by decapitation. Following a quick disinfection of the skin with ethanol (70%), the skull was opened and both cerebral hemispheres were carefully extracted. They were then placed in bench‐cold sterile phosphate‐buffered saline (PBS, pH 7.4), where cerebral cortices were dissected. The cortical tissue was then processed with a tissue chopper and enzymatically digested with Accutase (Gibco).

To determine the optimal coating strategy, cells were seeded on glass/ITO‐OEPC (⌀: 30 mm) coated with Geltrex (0.12 to 0.18 mg mL^−1^), poly‐D‐lysine (PDL; 0.1 mg mL^−1^), polyethyleneimine (PEI; 0.1%), or without any coating material. For further experiments, cells were seeded on PDL‐coated surface of glass/ITO‐ or glass/Au‐OEPC, or glass coverslips at 500 000 cells/sample in Dulbecco's Minimal Essential Medium (DMEM) with fetal bovine serum (FBS; 10%), non‐essential amino acids (100 µM) and penicillin‐streptomycin (100 U mL^−1^). to ensure cells will attach only to desired parts of the OEPC, metal rings (⌀: 10 mm) or glass rings (⌀: 6 mm) were placed on top of the *p–n* layer or the back electrode. After 3 h, the rings were removed, and cell medium was changed to the initial cortical medium (Neurobasal A, B‐27 supplement 2%, GlutaMAX 0.5 mM, basic fibroblast growth factor [bFGF] 5 ng mL^−1^, epidermal growth factor [EGF] 20 ng/mL, Normocin 100 µg mL^−1^), sustaining the culture for further 4 days. Subsequently, cells were provided with the continuation cortical medium (Neurobasal A, B‐27 supplement 2%, GlutaMAX 0.5 mм, bFGF 10 ng mL^−1^, Normocin 100 µg mL^−1^).

To inhibit excessive proliferation of glial cells, a mitosis inhibitor (final concentrations: 5‐fluoro‐2’‐deoxyuridine 100 nм, uridine 100 nм, cytosine‐β‐D‐arabinoside 10 nм) was added for 24 h to the continuation medium. Cell medium was then completely replaced, followed by additional changes of 50% of the media three times per week. The culture was sustained for an additional 10–11 days until maturation of neuronal networks.

### Animal Housing and Husbandry

All animal experiments were conducted with the approval of the local authorities (Austrian Federal Ministry of Education, Science, and Research; license number: 2021‐0.724.203) and reported using ARRIVE guidelines.

Adult male Sprague Dawley rats (10–14 week‐old; *n* = 41) were purchased from Charles River Laboratories (Sulzfeld, Germany) and housed in the animal facility of the Biomedical Research Institute at the Medical University of Graz under standard conditions with 12 h light/dark cycle and ad libitum access to food and water. Qualified personnel monitored the health status of the rats at least once a day.

Animals were randomly assigned to one of the three groups (Table S2, Supporting Information): parylene‐OEPC implantation and light stimulation (“Stimulation”; *n*  =  18), parylene‐OEPC implantation only (“Sham”, *n* = 15), or surgery and light stimulation, without implantation (“Light control”; *n* = 8). Four different time points for the experiments were defined. Acute stimulation was applied to immediately after the surgery (Stimulation, *n *= 3; Sham, *n* = 2). A delayed stimulation at 24 h post‐implantation was implemented in 11 rats (With durectomy: Stimulation, *n* = 3; Sham, *n* = 3. With intact dura: Stimulation, *n* = 3; Sham, *n* = 2). Seven animals underwent stimulation at 48 h (Stimulation, *n* = 3; Sham, *n* = 3; Light control, *n* = 1), and eighteen animals three weeks after implantation (Stimulation, *n* = 6; Sham, *n* = 5; Light control, *n* = 7).

One light control animal subdued a trauma during the surgery and was excluded from the experiment.

P0‐P1 pups for primary cell cultures were obtained from in‐house bred Sprague Dawley rats.

### Surgical Procedure

Animals were anesthetized with isoflurane (4%) followed by intraperitoneal administration of a fentanyl (0.04 mg kg^−1^), midazolam (0.8 mg kg^−1^), and medetomidine (0.4 mg kg^−1^) mixture (FMD). They were securely positioned on a stereotactic frame. Temperature stability was ensured using a heat blanket equipped with a rectal thermometer, and cling film was used to minimize body heat loss and prevent contamination from fur and skin. Scalp hair was removed with an electric trimmer, skin was disinfected using alcohol pads and povidone‐iodine solution. The skull was exposed by a longitudinal incision into the skin and subsequent periosteum removal. A hole (⌀: 5 mm) was drilled on the right parietal bone of the skull, with the anterior edge located ≈2 mm caudal to the bregma. After the bone flap removal, durectomy was performed in 3–5 steps. Special care was taken not to touch the exposed surface of the brain. Two additional holes (⌀: 0.5 mm) were drilled to place metal anchor screws that were tightened by 2 full turns.

Parylene‐OEPC was transferred from the wafer using a fine brush and placed onto the exposed cortex pre‐washed with physiological saline, with the photoactive layer facing cortical surface. Subsequently, the craniectomized hole was sealed with a custom 3D‐printed round implant made of medical‐grade transparent resin (⌀: 5 mm; thickness: 1 mm; BioMed Clear Resin, FormLabs, USA). The implant and screws were secured using dental cement, leaving the transparent window above the photoactive layer uncovered. The skin was sutured with absorbable material and the animals received subcutaneous injections of enrofloxacin (7.5 mg kg^−1^) to prevent post‐surgical infection and carprofen (2 mg kg^−1^; diluted in physiological saline) for pain control. Finally, anesthesia was reversed with subcutaneous administration of a mixture of flumazenil (0.105 mg kg^−1^) and atipamezole (0.63 mg kg^−1^) in physiological saline. Post‐surgical recovery was uneventful in all cases.

### Post‐Surgical Animal Welfare

Animals received enrofloxacin and carprofen as described above once daily for four days, unless the animal was sacrificed earlier. Animals were weighed on the day of surgery, 3 days post‐surgery, and before stimulation and sacrifice. Nest scores were evaluated on day 4, 10, and 17 post‐surgery through the analysis of images taken from the top and side of each cage. For the nest building control, two cages with two naive age‐matched male rats each were evaluated. Animal identifiers were then concealed and nest building quality was quantified on a scale from 0 to 4 as previously described in Schwabe et al.^[^ ^87^ ^]^

### Light Stimulation—Cell Culture

Cells in 6‐well plates were placed in a custom, opaque chamber 3D‐printed using fused deposition modeling to prevent artifacts caused by ambient light (Figure S13, Supporting Information). Ventilation slits on both sides enabled gas exchange. As a light source, each chamber was equipped with a high‐power, 660 nm matrix 10 W LED in the middle. The LED had a luminous flux of around 60 lm at 1 A. Due to the beam angle of 120°, the light source was mounted at a distance of 8 mm underneath the OEPC to ensure homogeneous illumination of the photoactive layer. The light output per area on the irradiated active layer was 8.21 mW mm^−^^2^. to minimize temperature fluctuations of the cell culture from the light source, a passive heat sink was used for dissipating heat.

Control of the high‐power LED was achieved using pulse‐width modulation (PWM) with a constant current source to ensure flexible control of the stimulation pulse ranging from µs to ms pulses. A second‐order low‐pass filter was employed to transform the high‐frequency PWM signal into an analog signal, with cut‐off frequencies adjusted to minimize fluctuations in the control signal for the respective stimulation protocols used. In order to make the stimulation protocols flexibly adaptable, an Arduino Uno was used to directly control the constant current source.

Cells were stimulated in standard incubator with a 30 min protocol with light pulses at 2 Hz (5 ms pulse and 495 ms interpulse for 10 cycles followed by 5 s break) or 20 Hz (2 ms pulse and 48 ms interpulse for 100 cycles followed by 5 s break). Sham controls cultured on OEPCs stayed in a dark chamber without stimulation. Positive controls were treated with 20 µm L‐glutamic acid for the same period. Culture plates were then removed from the chamber and left in the incubator for another 60 min before further processing.

### Light Stimulation—Animals

Animals underwent light stimulation or sham treatment, administered either acutely (immediately after the OEPC implantation), or at 24, 48 h, or three weeks following the surgery. All animals, except those undergoing acute stimulation, were anesthetized on stimulation day as described above and placed in the stereotactic frame. The wound was re‐opened, and a 700 mW 638 nm diode laser was positioned ≈1 cm above the implant (Figure S14, Supporting Information). Light conditions were kept at least 10× below the accepted class 4 laser safety limit for skin exposure, similarly to our previous works.^[^ ^27^, ^38^ ^]^ The laser light was focused over the visible *p–n* layer of the implanted OEPC or above the center of the transparent resin window in case of the light control group. Light stimulation was achieved through pulsed light (20 Hz stimulation, 2 ms light pulse, 48 ms interpulse length; ThorLabs) repeated continuously for 30 min. The sham animals were kept in darkness for the same duration.

Following the treatment and wound suture, anesthesia was reversed as described above. The animals were then left undisturbed in the home cage for 60 min. After this interval, they received an intraperitoneal injection of thiopental (200 mg kg^−1^) for terminal anesthesia. Upon respiratory arrest, the thorax was opened, and the rats were transcardially perfused with 4% (v/v) formaldehyde (FA) in PBS (pH 7.4) for 15–20 min. Subsequently, the skull was opened and the brain was carefully removed and immersed in 4% FA for 24 h at 4 °C for further fixation.

### Cytotoxicity Assay

Cytotoxicity in primary neuronal cultures was assessed using a colorimetric lactic dehydrogenase (LDH) assay using a commercial kit (Invitrogen CyQUANT LDH Cytotoxicity Assay) according to the manufacturer's instructions. Cell media was sampled from all cultures at DIV5, DIV10 as well as DIV14 before and after light/sham treatments, and transferred in triplicates to a transparent 96‐well plate. Absorbance was determined with a microplate reader (SPECTROstar Omega). Cytotoxicity was calculated as a percentage of LDH activity compared to the maximum LDH release control, as described by the product datasheet.

### Immunocytochemistry

List of used antibodies and dilutions is given in Table S3 (Supporting Information).

Cells underwent a brief wash in PBS and were then fixed in 4% (v/v) FA solution in diH_2_O for 15 min. After a wash in PBS with 0.3% Triton X‐100 (PBST; 3 × 5 min), 5% (v/v) normal goat serum (NGS) in PBST was applied for 60 min at room temperature to block for unspecific binding. The cells were then incubated overnight at 4 °C in with primary antibody. The next day, the samples were washed in PBST (3 × 5 min) and incubated with a secondary antibody for 60 min at room temperature. After the final wash in PBST (3 × 5 min), OEPC with cells were covered using mounting media with DAPI (FluoroShield) and 18 × 18 mm square glass coverslips, secured with transparent nail polish.

### Tissue Sectioning

Frozen sections of 20 µm were prepared at a cryomicrotome after cryopreservation of the fixed tissue with 30% sucrose, quick freeze using a cryospray and finally embedding in a water‐soluble embedding media (OCT Compound, Tissue‐Tek). Brain samples for paraffin sections were dehydrated in a tissue processor (Tissue‐Tek VIP 5 Tissue Processor, Sakura). After paraffin embedding, 2 µm sections were prepared in a standard histology microtome. From each brain, fifteen series of sections encompassing five regions was acquired: a) bregma +1.92 to 0.48, b) bregma 0.00 to −1.28, c) bregma −2.56 to −3.36, d) bregma ‐4.08 to −4.72 and e) bregma −4.92 to −5.68. Sections in (a) and (b) corresponded to brain parts localized rostrally to the implant (containing motor cortex and partially somatosensory cortex), while sections in (c)–(e) were situated below the implant (spanning over the somatosensory cortex and parts of visual cortex).

### Immunofluorescence of Cryosections

List of used antibodies and dilutions is given in Table S3 (Supporting Information).

Cryosections were post‐fixed with ice‐cold methanol for 10 min, then washed in diH_2_O (3 × 3 min). Next, 10 mm sodium citrate buffer (pH 6.0) was boiled in the microwave and the samples were soaked for 3 min on the bench for antigen retrieval. After another wash in diH_2_O (3 × 3 min), samples were encircled with a hydrophobic PAP pen (Merck) and placed in a humidity chamber filled with tissue paper soaked in distilled water. A 5% NGS in PBST blocking solution was applied for 30 min, which was followed by incubation with the primary antibody in the humidity chamber overnight at 4 °C. Samples were then washed in PBST (3 × 5 min) and incubated with the secondary antibody for 60 min at room temperature. From this point onward, the samples were kept in the dark to prevent fluorophore bleaching. After a wash in PBST (3 × 5 min), object slides with brain sections were covered using mounting media with DAPI (FluoroShield) and glass coverslips, secured additionally with transparent nail polish.

### Histological and Immunohistochemical Staining of Paraffin‐Embedded Sections

One complete series of paraffin‐embedded brain sections was stained with thionine to mark the Nissl substance of neurons. For this purpose, sections from all animals were deparaffinized and rehydrated through treatment with xylene (2 × 3 min) and ethanol gradient (100% 1 × 3 min, 90% 1 × 3 min, 70% 1 × 3 min, and 50% 1 × 3 min). After washing with distilled water, the sections were immersed in a thionine solution at room temperature for 20 s. Following tissue dehydration in an increasing gradient of ethanol (50% 1 × 3 min, 70% 1 × 3 min, 90% 1 × 3 min, and 100% 1 × 3 min) and xylene (2 × 3 min), object slides were coverslipped using mounting media (Sakura). These sections were then used for orientation in the brain during the quantification of c‐Fos staining.

Immunohistochemistry (IHC) was performed on five series of brain sections to investigate c‐Fos and markers of glial and immune cells. Tissue sections from all animals were used in c‐Fos analysis. For the markers of glial and immune cells, IHC was conducted on sections from 9 animals (*n* = 3 per group; OEPC with stimulation; OEPC only, light only).

Sections underwent deparaffinization and rehydration steps as described above. After immersion in 100% ethanol, the tissue was treated with a 0.67% H_2_O_2_ solution in methanol for 30 min to quench endogenous peroxidase activity. Following a wash in distilled water (2 × 5 min), samples were transferred to a decloaking chamber and soaked in sodium citrate buffer (10 mм, pH 6.0). Antigen retrieval was performed at 95 °C for 20 min under constant pressure.

Subsequently, the sections were washed in PBST (2 × 5 min), circumscribed with a hydrophobic PAP pen (Merck), and placed in a humidity chamber. Depending on the secondary antibody host, 5% NGS or 5% horse serum in PBST was added for 60 min at room temperature to block unspecific binding sites, and then incubated with primary antibody overnight at 4 °C. Samples were again washed in PBST (3 × 5 min) and incubated with secondary antibody for 1 h at room temperature. Following further PBST wash (3 × 5 min), the avidin‐biotin complex was added for 30 min at room temperature to amplify the signal. The sections were then washed PBST (3 × 5 min) and color was developed using a chromogen (3,3’‐diaminobenzidine; DAB) solution for 1–3 min. After a wash in diH_2_O (2 ×5 min), the sections were briefly (1–2 s) immersed in hematoxylin solution, left for 5 min under running distilled water, and subsequently dehydrated in an increasing gradient of ethanol and xylene (50% 1 × 5 min, 70% 1 × 5 min, 90% 1 × 5 min, 100% 1 × 5 min), a 1:1 mixture of xylene and ethanol (100%; 1 × 5 min) and xylene (2 × 5 min). Finally, they were coverslipped using xylene‐based mounting media (Sakura).

Double and triple immunofluorecent (IF) staining procedures were performed in the same manner as described above, excluding the endogenous peroxidase blocking step. Both primary and secondary antibodies (Table S3, Supporting Information) were diluted in the 5% NGS blocking solution. After a thorough wash in PBST (3 × 5 min), object slides were coverslipped using aqueous mounting media with DAPI (FluoroShield). Nail polish was applied at the corners to secure the coverslips in place. All steps from the secondary antibody incubation onward were performed in darkness to prevent fluorophore bleaching.

### Image Acquisition and Analysis

12‐bit images of immunocytochemistry (ICC) were acquired using a confocal microscope (Nikon A1R) under 200× magnification, with an image size of 1024 × 1024 pixels, a pixel dwell time of 0.5 µs, and an optical resolution of 470 nm. Pixel values from four scans were averaged for the final image. For glass/ITO‐OEPC, five separate images were obtained for each sample at the level of the *p–n*layer of the OEPC, with one in the middle and four at each arm of an imaginary compass rose. This pattern was also applied to the cell culture on glass coverslips in a control experiment. Regarding glass/Au‐OEPC, three images were captured for each of the device regions: inner part of the *p–n* layer, the outer part of the *p–n* layer, and back electrode.

Image analysis was performed using Fiji software (National Institutes of Health; Maryland, USA). Images in the native Nikon format (ND2) were opened with the Fiji Bio‐Formats plugin.^[^ ^88^ ^]^ Teo calculate the percentage of c‐Fos^+^ cells, the threshold was initially set for the DAPI channel using an automatic Moments threshold and the number of nuclei was determined using the Measure Particles tool with specific parameters (particle size: 25–200 µm; circularity: 0.2–1.0). The calculated nuclei were automatically added to the Region of Interest (ROI) manager. The green channel image was processed in a similar manner, with the previously saved ROI added to quantify precisely the same regions as in the DAPI image. The proportion of particle numbers calculated in the green channel relative to those in the blue channel provided the percentage of c‐Fos^+^ cells. The average values from images from each area served as a single data point for the statistical analysis.

For the analysis of the mean grey value across the entire image, the green channel image, containing c‐Fos staining, was selected. The grey value threshold was set using the automatic Fiji‐innate method (Moments; threshold mean: 198.84; threshold standard deviation: 75.95). The mean grey value of each thresholded image was then measured and averaged for each sample.

Images from DAB‐stained sections were captured using a slide scanner (Aperio Scan Scope AT, Leica, Germany). Scans of object slides parts containing brain sections were captured at 200× (c‐Fos) and 400× (glial and immune cell markers) magnifications and saved in the Aperio ScanScope Virtual Slide (SVS) format.

The analysis of DAB staining of c‐Fos in brain sections was conducted using QuPath software (v0.4.3). In each section, the somatosensory cortex, entorhinal cortex, and hippocampus were delineated in the ipsi‐ and contralateral hemispheres. Cells were considered positive if the value of mean optical density within the nucleus reached a threshold of 0.2 in the DAB channel. The hematoxylin channel was used for calculating the total cell number, employing the optical density threshold of 0.05. For each region of interest, the percentage of positive cells was calculated. Averages were determined for each object slide with sections located beneath the surgical site.

Double and triple IF staining of c‐Fos with various cellular markers was inspected in slices located beneath the implanted/craniectomy site. For each, one image of the ipsilateral and contralateral somatosensory cortex and hippocampus was captured using a confocal microscope with the same parameters as described for ICC.

### Preparation of Explanted Flexible OEPC for Scanning Electron Microscopy (SEM)

All parylene‐OEPC were carefully removed from either the brain surface or the transparent resin window during organ collection.

Initially, the samples were fixed (2% paraformaldehyde, 2.5% glutaraldehyde, 0.1 м cacodylate buffer (CB), pH 7.4) and then transferred to CB for 30 min (0.1 м, pH 7.4). Then, CB was removed, and OsO_4_ solution (2% OsO4 in 0.1 м CB) was pipetted onto the samples. After a 30 min incubation in darkness, the OsO_4_ solution was exchanged for CB. Samples not stained with osmium remained in CB for the duration of the process.

Subsequently, parylene‐OEPC was dehydrated in an increasing ethanol gradient (30% 1 × 15 min, 50% 1 × 15 min, 70% overnight, 80% 1 × 15 min, 90% 1 × 15 min, 96% 1 × 15 min and 100% 2 × 7 min). For sample drying, a 1:1 mixture of ethanol (100%) and hexamethyldisilazane (HDMS) was applied (1 × 5 min), followed by the exchange for pure HDMS (1 × 5 min). Dried samples were placed in a desiccator overnight. Finally, parylene‐OEPC was sputter‐coated with carbon to render them conductive. Image acquisition was performed with a scanning electron microscope (Sigma 500 VP, Zeiss) using the Zeiss SmartSEM imaging software.

### Functional Assessment of Flexible OEPC

The functionality of flexible OEPC devices was verified by a transient voltage measurement technique. The whole device was submerged in PBS, one Ag/AgCl electrode enclosed in a syringe equipped with a Chipquik SMDTA30 grey tip was put in close proximity of the center of the *p–n* pixel, the other Ag/AgCl electrode was placed directly in the PBS bath at a distance of 4 mm from the syringe electrode. The photogenerated voltage measured between the two electrodes was captured by an oscilloscope (Picoscope 5243B). The illuminating light‐emitting diode (LED, Thorlabs M660L4, 660 nm, 1 mW mm^−2^) was driven using a Thorlabs DC2200. The driver pulse generator was used to trigger the oscilloscope. The light intensity was calibrated using a Thorlabs SM1PD1A photodiode. Device functionality was verified both after fabrication and subsequent to explantation in the in vivo experiment.

### Temperature Measurements

To assess possible heating effects of the laser on brain tissue, a 1:1 scale brain model was fabricated as depicted in Figure S12A (Supporting Information). A 1 % (w/v) Phytagel solution in deionized water was heated to 85 °C and stirred for 30 min. The mixture was then cooled down to room temperature and subsequently frozen at −20 °C to fabricate a brain‐inspired hydrogel. The hydrogel was colored with 0.1% (w/v) methylene blue to enhance visual contrast and was poured into a 3D‐printed mold based on MRI scans of a rat brain. The OEPC was placed on top of the hydrogel brain phantom. Additionally, a skull phantom was 3D‐printed using stereolithography to accurately mimic the anatomical structure of a rat's skull. A cranial window made of medical‐grade transparent resin (described above) was placed on top of the OEPC.

The experimental setup included a laser positioned 10 mm above the OEPC in an incubator at 37 °C to replicate the conditions of the in vivo experiments. The humidity was maintained at 100% to prevent drying out of the brain phantom. Heat distribution post‐stimulation was assessed using thermographic measurement (Thermo Tracer TH7800N). The measurements were conducted after an initial incubation period of 2 h, subsequent 30 min of stimulation according to the in vivo experimental protocol, and a resting phase of 10 s after opening of the incubator. The same protocol was applied without laser stimulation as a control measurement. The cranial window was removed right after opening the incubator in all experiments.

### Statistics

Statistical analysis of all numerical data was performed using R Statistical Software (v4.2.0, 2022‐04‐22)^[^ ^89^ ^]^ and its relevant packages: dplyr (v1.1.4, 2023‐11‐17),^[^ ^90^ ^]^ tidyverse (v2.0.0, 2023‐02‐22),^[^ ^91^ ^]^ car(v3.0‐13, 2022‐05‐02),^[^ ^92^ ^]^ ggplot2 (v 3.4.4, 2023‐10‐12),^[^ ^93^ ^]^ ggrepel (v0.9.4, 2023‐10‐13),^[^ ^94^ ^]^ and ggpubr (v0.6.0, 2023‐02‐10).^[^ ^95^ ^]^ The normality of data distribution was assessed using the Shapiro‐Wilk test. Variances were compared using the F‐test for two groups and Levene's test for three or more groups. Details of additional statistical tests utilized in the analyses can be found in Table S4 (Supporting Information). Significance for all tests was considered at *p* < 0.05.

## Conflict of Interest

The authors declare no conflict of interest.

## Author Contributions

M.N., M.J., S.P., K.K., T.R., V.Đ., E.D.G, R.S., and M.Ü. performed conceptualization. M.N., M.J., T.S., A.O., M.P., R.R., J.F., D.Z., K.K., T.R., V.Đ., E.D.G., R.S., and M.Ü. performed methodology. M.N., M.J., T.S., A.O., M.P., R.R., J.F., D.Z., V.Đ., E.D.G., R.S., and M.Ü. performed validation. M.N. did formal analysis. M.N., M.J., T.S., M.P., R.R., and J.F. performed investigation. M.N. performed data curation. M.N., M.J., A.O., M.P., R.R., and V.Đ. wrote the original draft. All authors wrote, reviewed and edited the final manuscript. M.N. performed visualization. S.S., K.K., T.R., and M.Ü. acquired funding. S.S, K.K., T.R., V.Đ., E.D.G., R.S., and M.Ü. supervised the project.

## Supporting information

Supporting Information

## Data Availability

The data that support the findings of this study are available from the corresponding author upon reasonable request.
