# Supplementary material for: Light‐Controlled Electric Stimulation with Organic Electrolytic Photocapacitors Achieves Complex Neuronal Network Activation: Semi‐Chronic Study in Cortical Cell Culture and Rat Model
Source: Adv Healthc Mater. 2024 Aug 13;13(29):2401303. doi: 10.1002/adhm.202401303 (PMC11582505; doi:10.1002/adhm.202401303)
Supplement: Supplementary file 1 — Supporting Information [file ADHM-13-0-s001.pdf]

ADVANCED HEALTHCARE MATERIALS

Supporting Information

for *Adv. Healthcare Mater.*, DOI 10.1002/adhm.202401303

Light-Controlled Electric Stimulation with Organic Electrolytic Photocapacitors Achieves Complex Neuronal Network Activation: Semi-Chronic Study in Cortical Cell Culture and Rat Model

*Marta Nowakowska, Marie Jakešová, Tony Schmidt, Aleksandar Opančar, Mathias Polz, Robert Reimer, Julia Fuchs, Silke Patz, Daniel Ziesel, Susanne Scheruebel, Karin Kornmueller, Theresa Rienmüller, Vedran Đerek, Eric D. Głowacki, Rainer Schindl and Muammer Üçal**

Supporting Information

Light-Controlled Electric Stimulation with Organic Electrolytic Photocapacitors Achieves Complex Neuronal Network Activation: Semi-chronic Study in Cortical Cell Culture and Rat Model

*Marta Nowakowska, Marie Jakešová, Tony Schmidt, Aleksandar Opančar, Mathias Polz, Robert Reimer, Julia Fuchs, Silke Patz, Daniel Ziesel, Susanne Scheruebel, Karin Kornmueller, Theresa Rienmüller, Vedran Đerek, Eric D. Głowacki, Rainer Schindl, and Muammer Üçal**

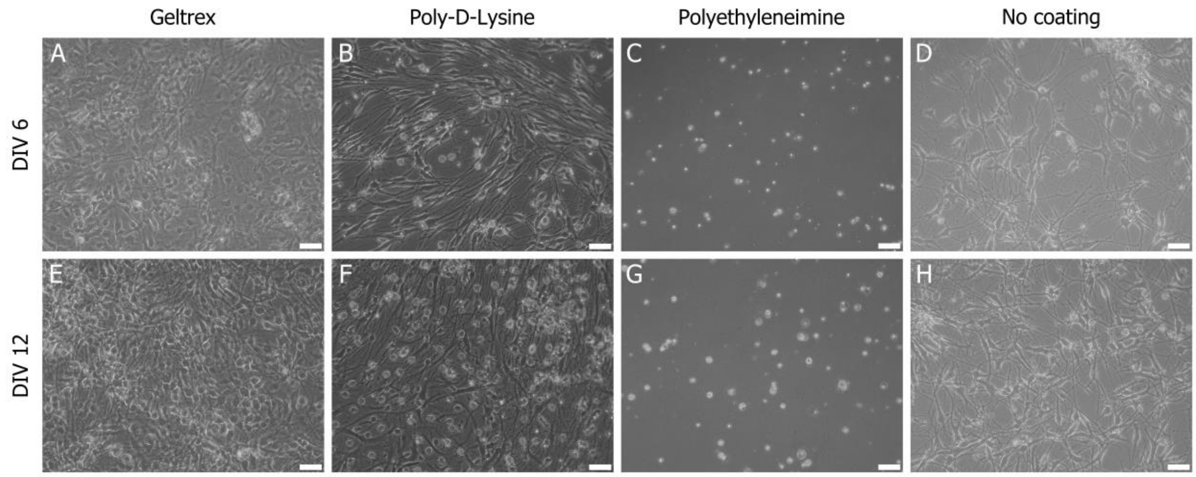

Figure S1. Comparison of various coating strategies on the glass/ITO-OEPC. (A-D) Representative photomicrographs of rat primary cortical cell culture acquired above the p-n layer of the device at day *in vitro* (DIV) 6. (A) OEPC coated with Geltrex, (B) poly-D-lysine, (C) polyetheleneimine, or (D) without any coating. (E-H) Representative photomicrographs of rat primary cortical cell culture acquired above the p-n layer of the device on DIV12. (E) OEPC coated with Geltrex, (F) poly-D-lysine, (G) polyetheleneimine, or (H) without any coating. Scale bar: 50 µm

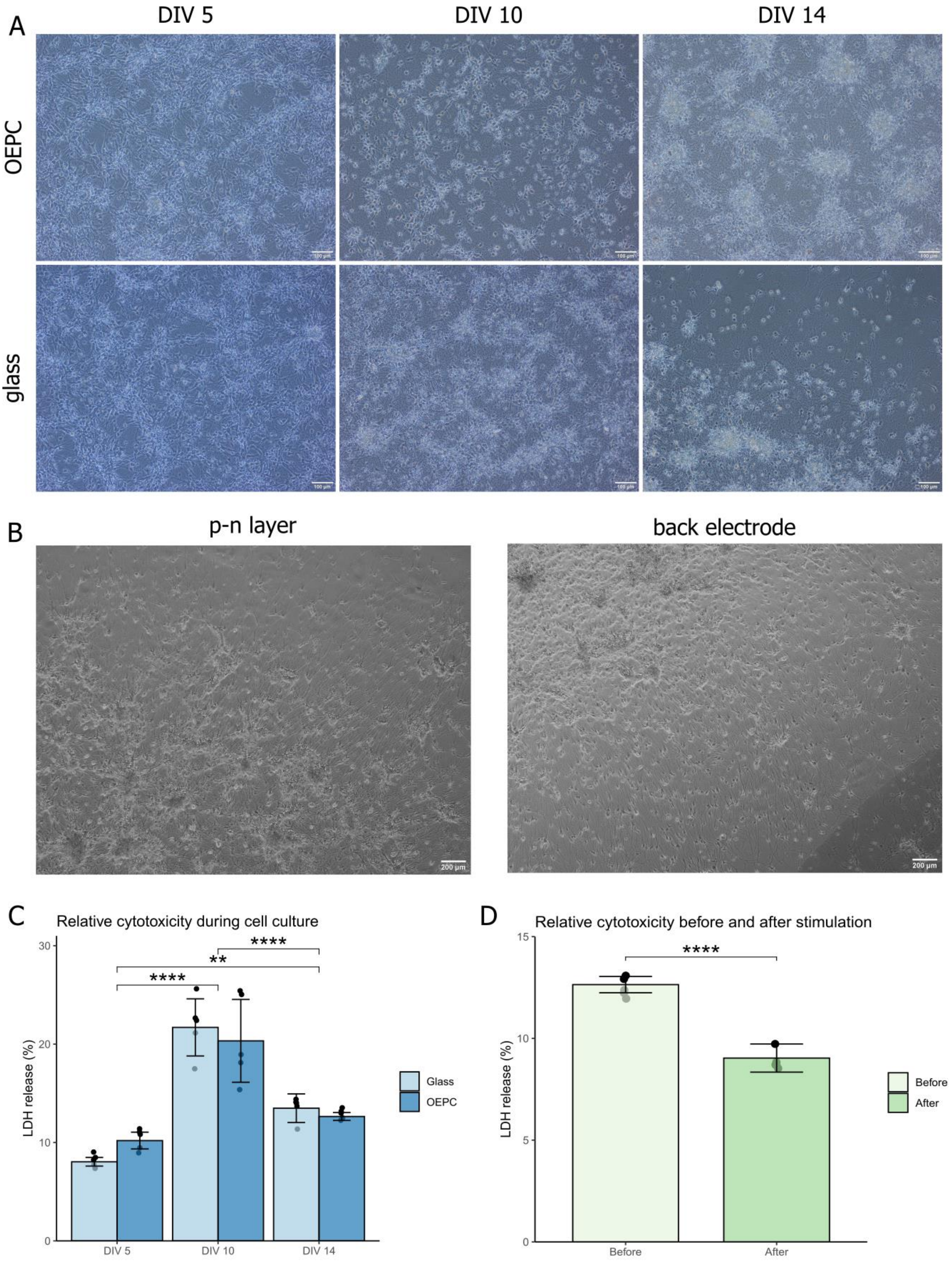

Figure S2. Primary cortical cell survival on poly-D-lysine coated glass/Au-OEPC. (A) Representative photomicrographs of different parts of OEPC and a glass cover slip at different time points of the cell culture. Cell cultivated on top of the OEPC (upper row) showed high density throughout the culture, comparable to the density of cells cultivated on glass (lower row). At day *in vitro* (DIV) 14 visible clusters of cell debris. (B) Comparison between the cells seeded on top of the p-n layer and back

electrode at DIV14. Comparable cell density and complexity of neuronal networks. (C) Relative cytotoxicity of the material measured in terms of LDH absorbance in cell culture medium sampled at DIV5, DIV 10 and DIV14. (D) Relative cytotoxicity of the treatment measured in terms of LDH absorbance in cell culture medium sampled before and after stimulation. ** $p < 0.01$; **** $p < 0.0001$. Scale bar: (A): 100 µm, (B): 200 µm.

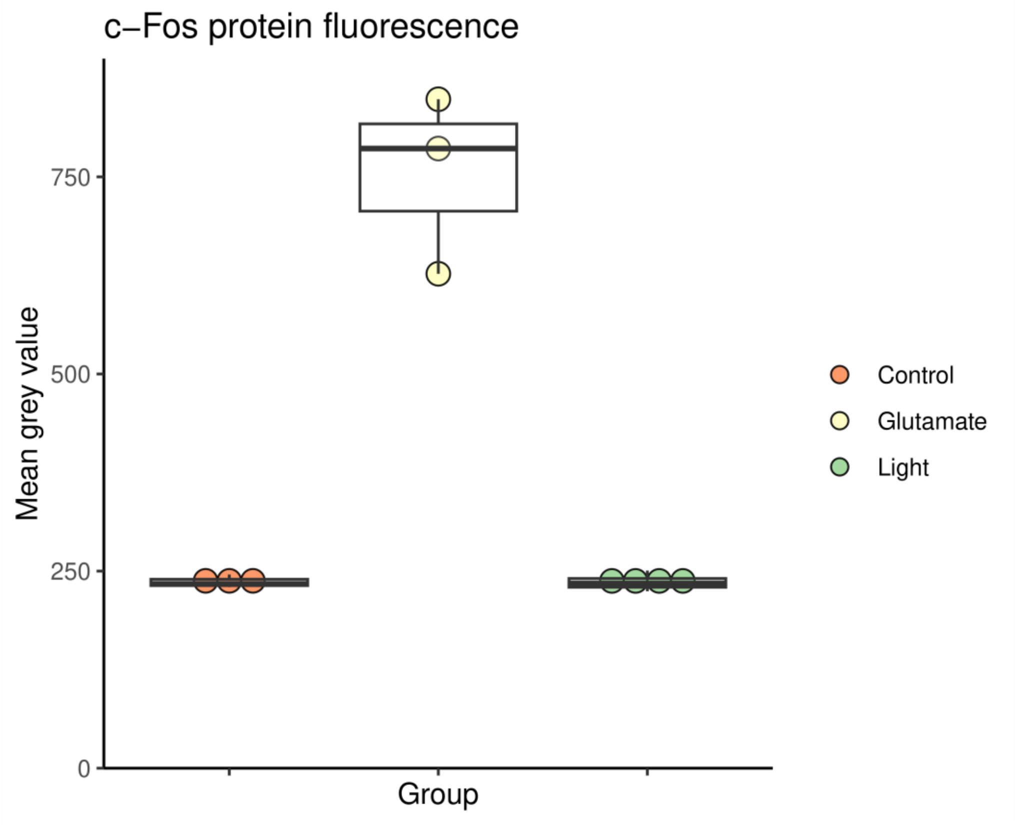

Figure S3. **Mean grey value of c-Fos immunostaining in rat primary cortical cell culture on glass cover slip.** To exclude a potential effect of the light treatment itself, cells grown on glass were subjected to either control conditions (“Control” – cells left in darkness; “Glutamate” – cells left in darkness with addition of 20 µM L-glutamic acid) or light treatment (“Light” – cells treated with red light at 20 Hz with 2 ms pulse). The mean grey value of the control and light treated cells did not differ between those groups, while staining of glutamate-treated cells demonstrated a visibly more intense staining.

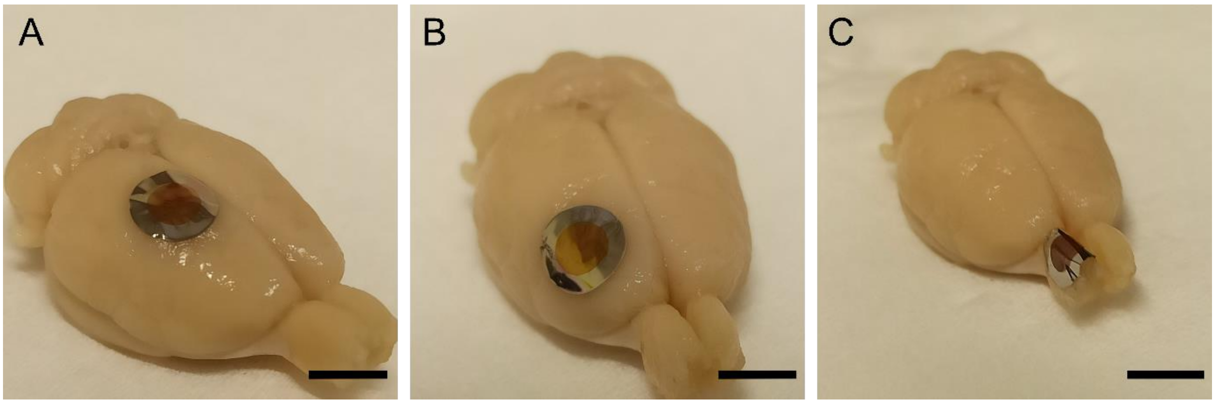

Figure S4. **OEPC placed on three surfaces of perfused rat brain shows its high flexibility.** (A) OEPC on top of the parietal cortex, relatively flat part close to the actual location of the OEPC during the experiment, (B) OEPC on top of the frontal cortex, with a more pronounced curvature, and (C) OEPC wrapped around the olfactory bulb, a narrow region with high curvature.

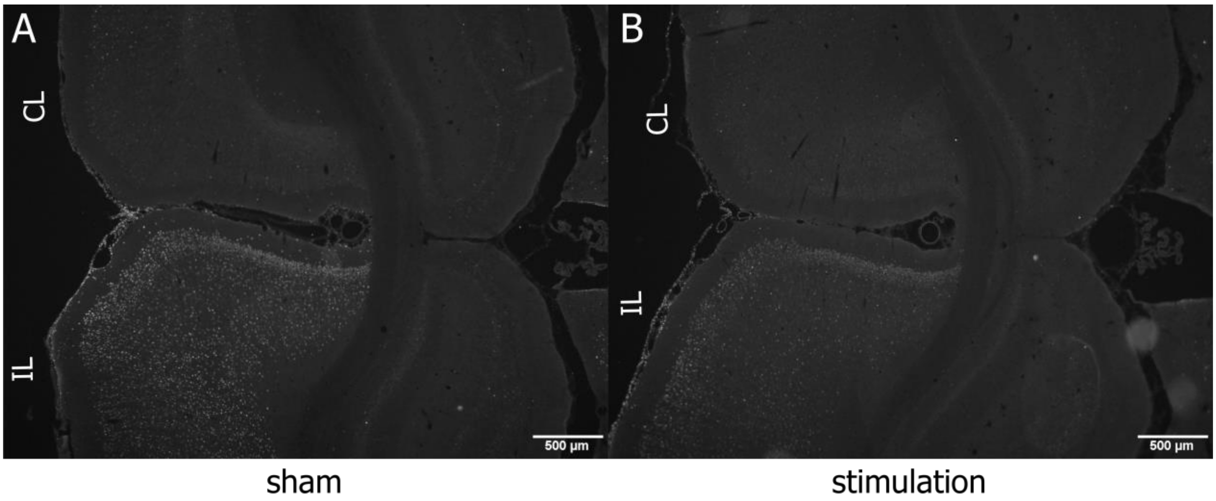

Figure S5. c-Fos immunoreactivity in coronal brain sections following acute stimulation. 30 minute 20 Hz light treatment was performed during the surgery, immediately after OEPC implantation. Extensive c-Fos expression was observed across the entire cortex of the ipsilateral (IL) hemisphere, regardless of the treatment, whereas only individual c-Fos (+) cells were noted in the contralateral (CL) cortex.

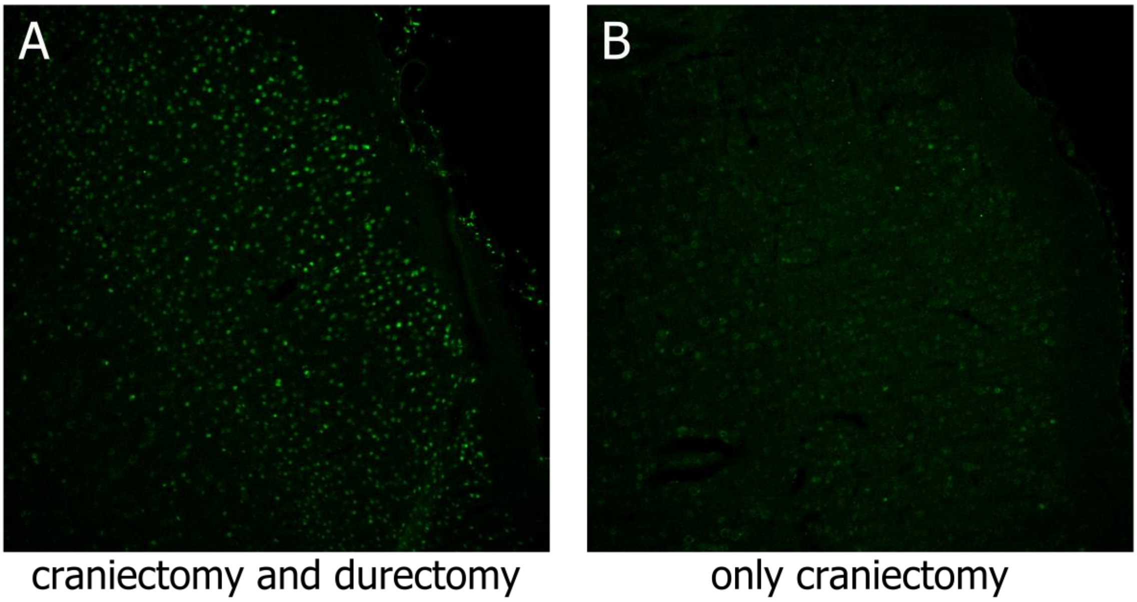

Figure S6. c-Fos immunoreactivity increases following durectomy. (A) c-Fos expression in the ipsilateral parietal cortex 60 minutes after craniectomy with subsequent durectomy. (B) c-Fos expression in the ipsilateral parietal cortex 60 minutes following only craniectomy. Extensive c-Fos expression was observed in the cortex only when dura mater was removed, while the animal with intact dura mater displayed individual c-Fos (+) cells.

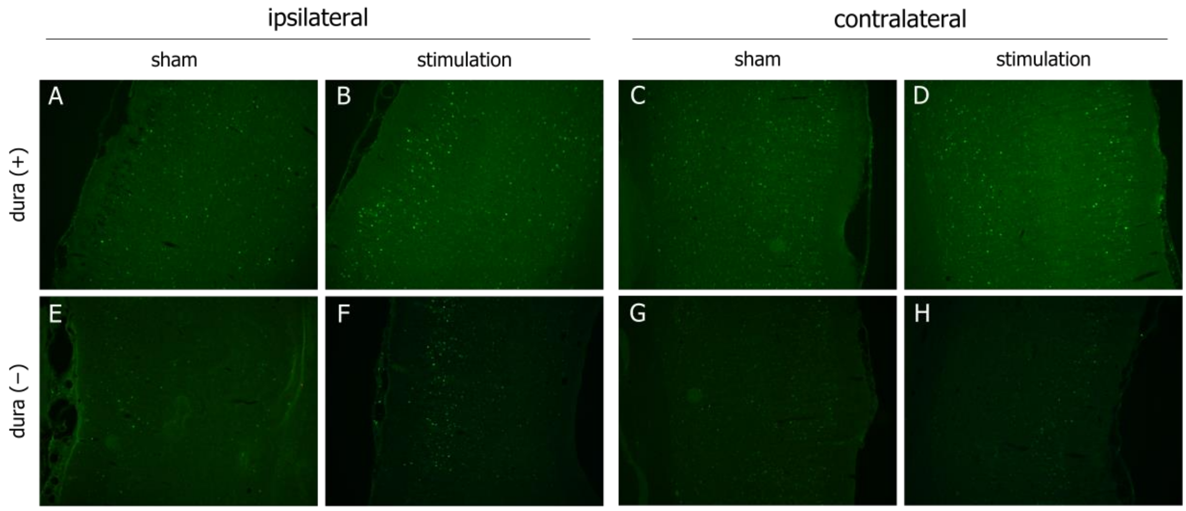

Figure S7. c-Fos immunoreactivity in the parietal cortex in animals subjected to the 30 minute OEPC stimulation 24 hours post-implantation. (A-D) c-Fos immunoreactivity after stimulation OEPC were positioned on top of the parietal cortex with dura mater left intact. (E-H) c-Fos immunoreactivity after stimulation OEPC were positioned on top of the parietal cortex following dura removal. In the ipsilateral cortex, more c-Fos (+) cells were observed following the stimulation compared to the sham treatment in both animals without durectomy (A, B) and after durectomy (E, F). The signal was mostly discernible in the layers II/III of the parietal cortex. No visible differences in c-Fos expression were observed on the contralateral side, regardless of the status of the dura mater (C, D, G, H).

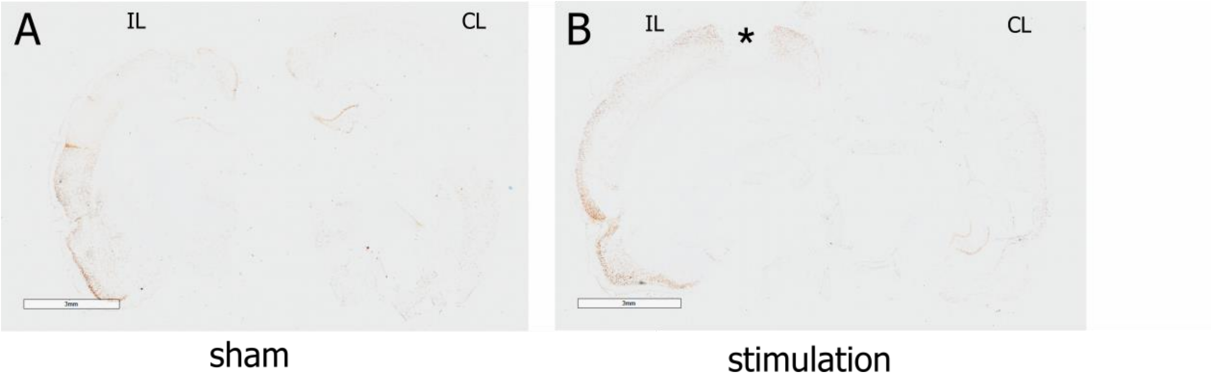

Figure S8. c-Fos immunoreactivity in coronal brain sections following 30 minute OEPC stimulation performed 48 h post-implantation. (A) Widespread c-Fos expression throughout the cortex in the ipsilateral (IL) hemisphere in sham animals. (B) (A) Widespread c-Fos expression throughout the cortex in the ipsilateral (IL) hemisphere in stimulated animals. Asterisk indicates a site of cell loss due to surgical trauma.

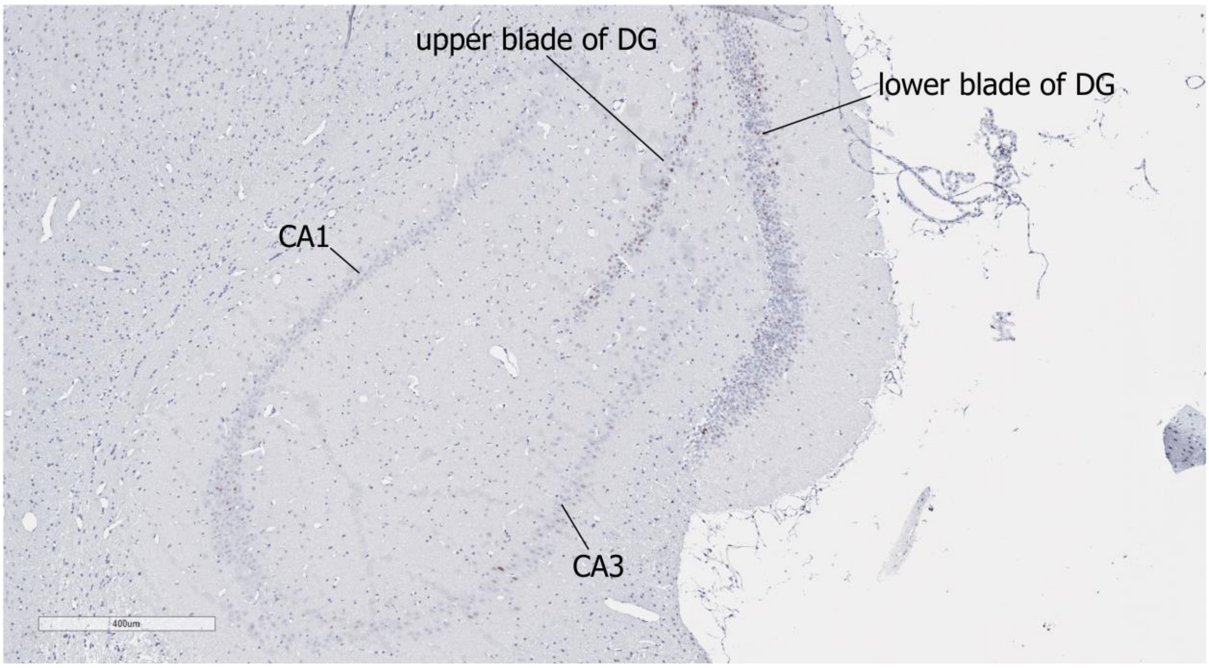

Figure S9. c-Fos immunoreactivity in the hippocampus. Following three weeks implantation, a 30 minute 20 Hz OEPC stimulation caused an increase in c-Fos^{+} cells within the hippocampus, particularly pronounced in the dentate gyrus (DG), especially in its upper blade. Less c-Fos^{+} cells were observed in Cornu Ammonis (CA)3 with only single positive cells present in CA1. Scale bar: 400 µm

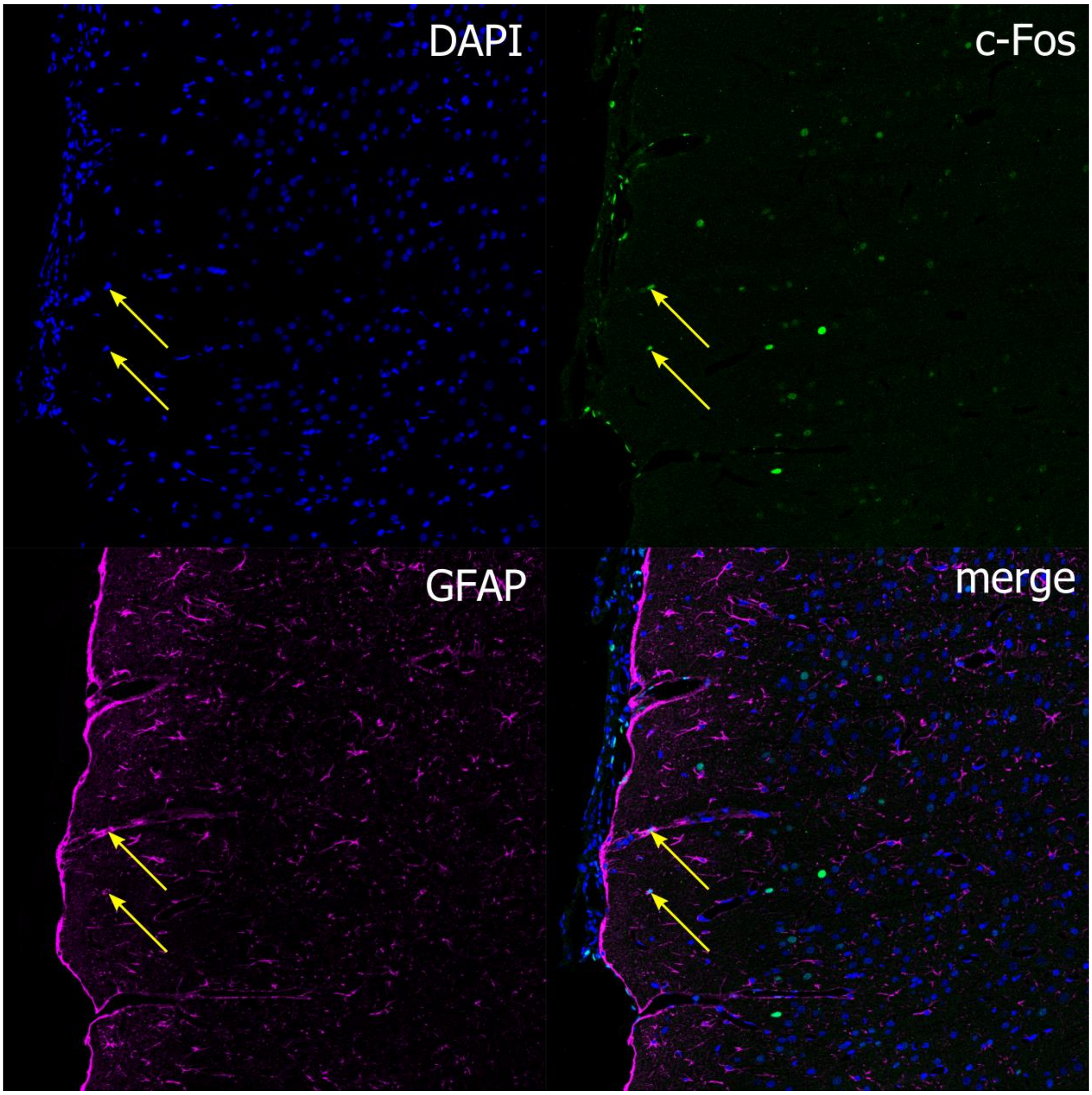

Figure S10. Exceptional c-Fos/GFAP co-expression in the cortical layer I. Double immunofluorescent staining of c-Fos (green) and GFAP, marker of astrocytes (magenta) with additional DAPI counterstaining of nuclei (blue) in the animal referenced in Figure 7. Arrows indicate cells co-expressing c-Fos and GFAP observed in the cortical layer I, close to the surgical site.

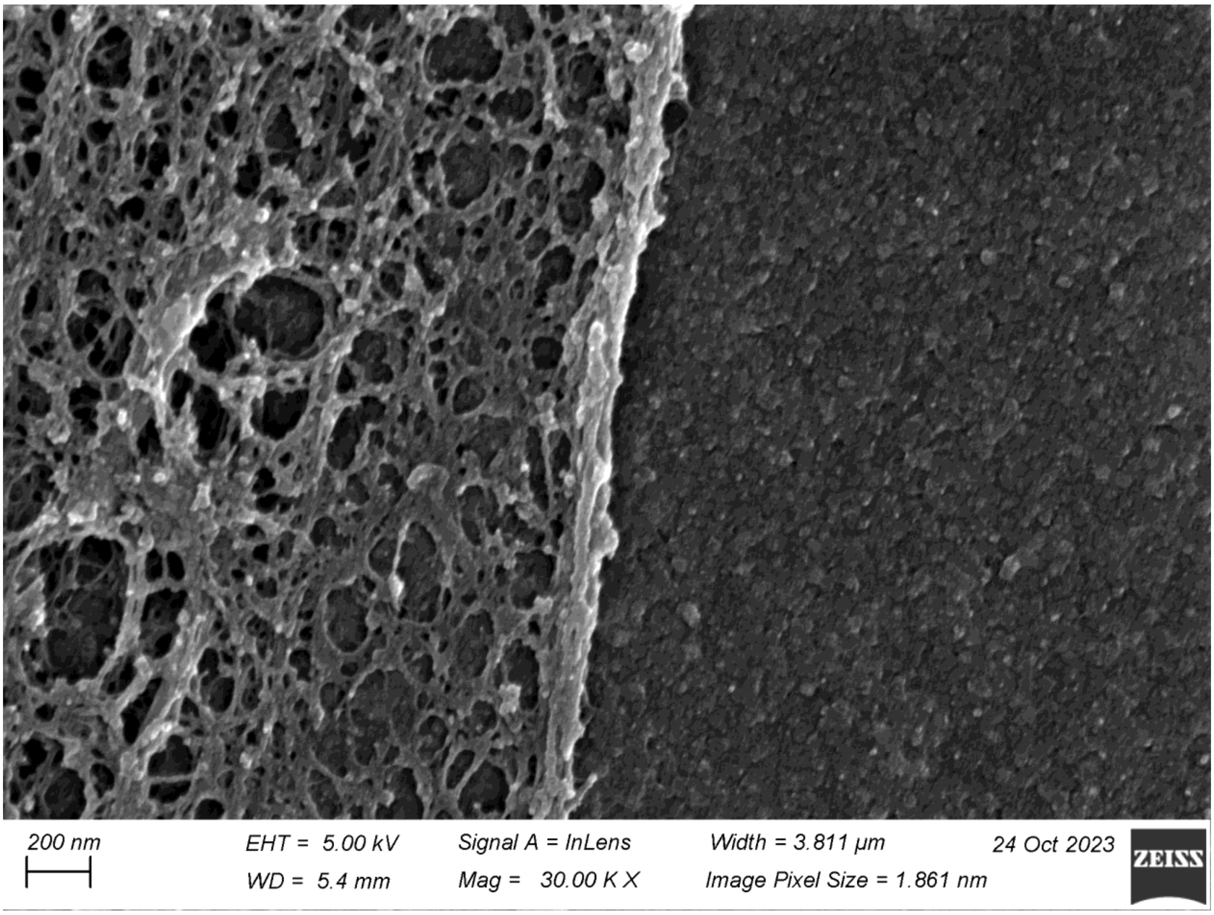

Figure S11. **Representative SEM photomicrograph of the surface of the p-n layer with attached tissue.** Visible smoothening of the p-n layer next to the tissue. Similarly to the OEPC free of biological material (Figure 8A-D), no signs of delamination or other damage to the surface was observed.

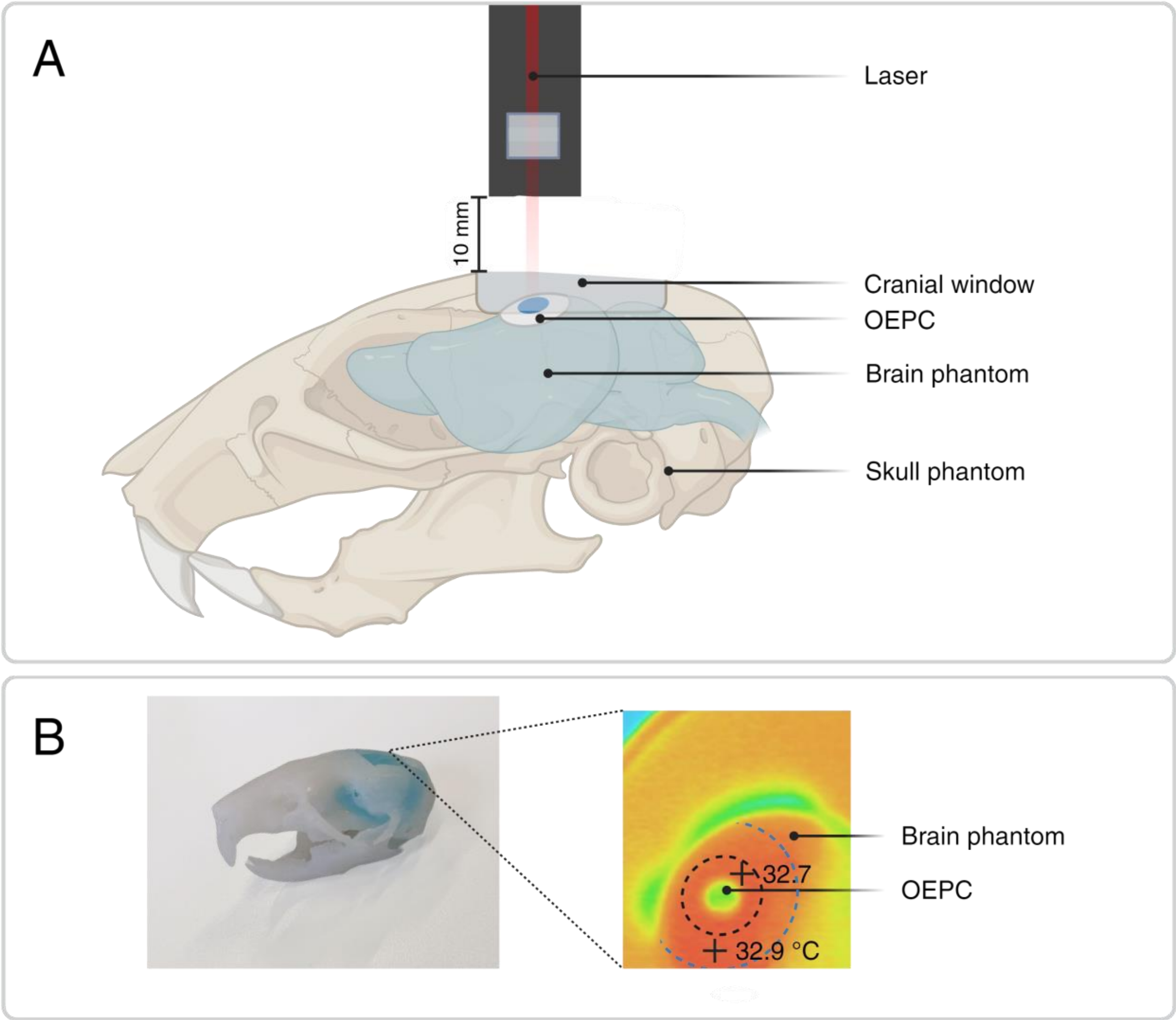

Figure S12. Temperature measurements following laser light stimulation. (A) Schematic of the experimental setup showing the stimulation laser, conformal OEPC, Phytagel brain phantom, and skull phantom with the cranial window. (B) Surface temperature (top view) was measured 10 s after stimulation using 2 ms pulses of 700 mW 638 nm laser for 30 minutes. Cranial window was removed before measurement but its position is highlighted with a black dotted line.

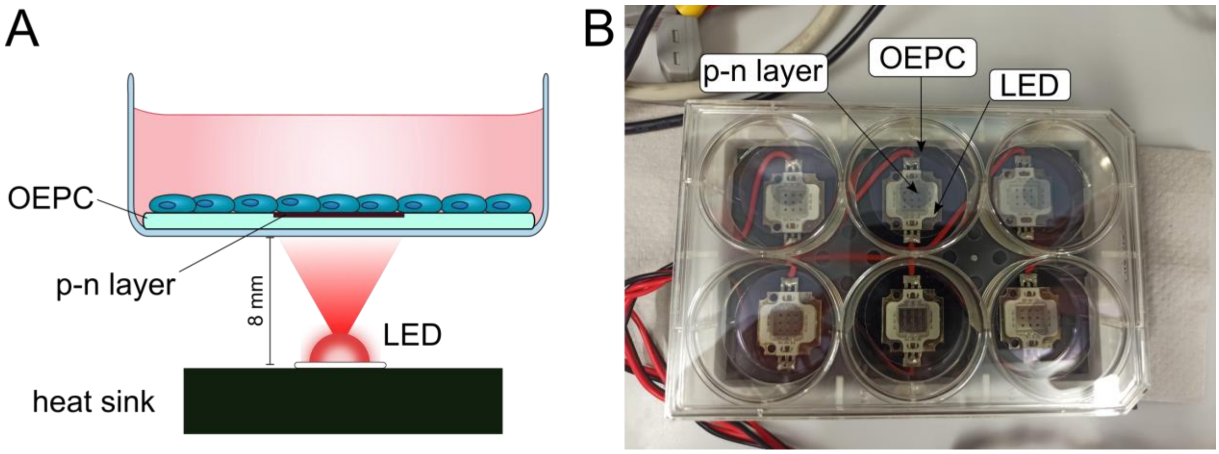

Figure S13. **Experimental setup for the OEPC stimulation *in vitro*.** (A) Schematic representation of the cell culture during the stimulation, side view. Primary cortical cells were grown on the surface of OEPC placed in 6-well plates. For the stimulation, the well plates were placed in custom-made chamber with LED located underneath. Metal heat sink is used to dissipate the temperature rise during the LED duty cycle. (B) Top view of the well plate with 6 OEPC devices placed into the chamber with LED located on the heat sink. Icons from bioicons are dedicated to the public domain under CC0 license.

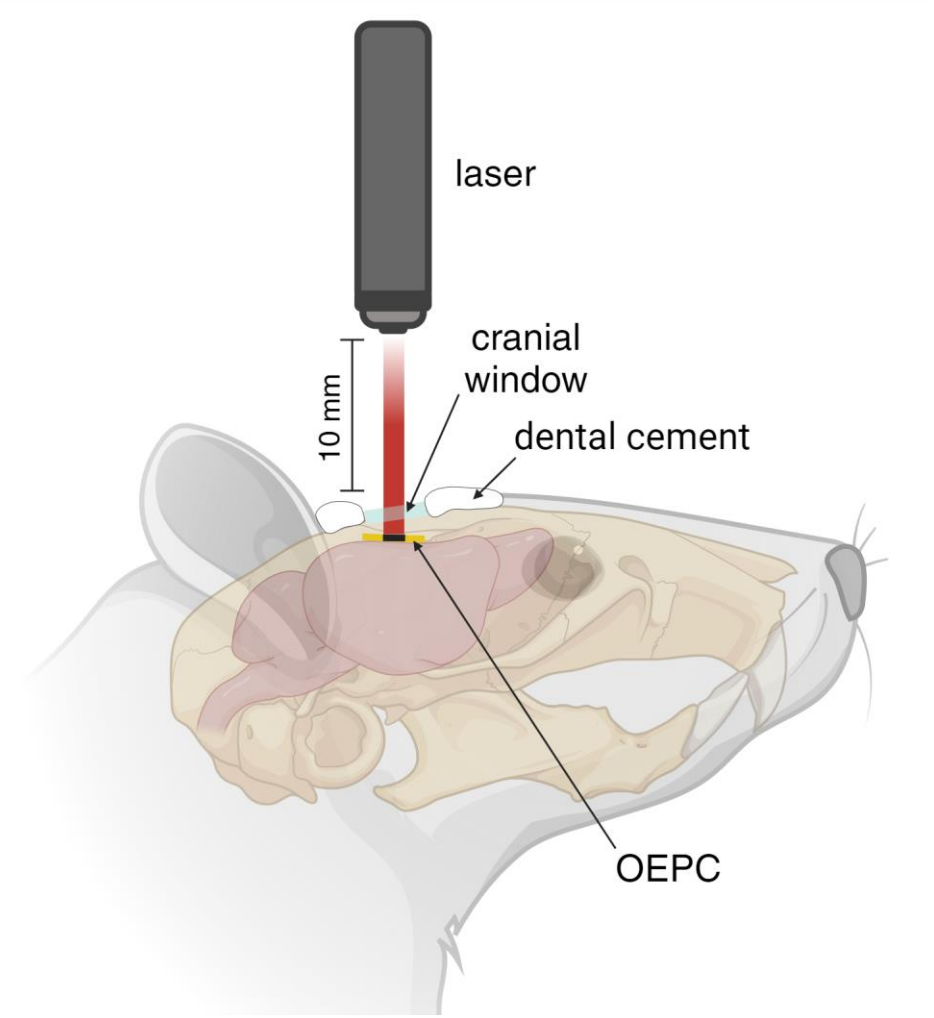

Figure S14. Schematic representation of the experimental setup for the OEPC stimulation *in vivo*. While the animal's head is stabilized in the stereotactic frame, the laser is placed approximately 10 mm above its head. The wound is reopened and the laser light is centred above the visible p-n layer (or the central part of the cranial window in the light control animals). Created with BioRender.com

Table S1. List of the materials, equipment and software used in the study.

Item	Reference
**OEPCs fabrication - *in vitro* application**	
Materials:	
ITO-covered glass coverslips	Kintec, Hong Kong (d = 30 mm, thickness 0.4 mm, sheet resistance 12-15 ohm/sq)
Phthalocyanine (H_2Pc)	Sigma Aldrich/Merck, 931985
N,N'-dimethyl-3,4,9,10-perylenetetracarboxylic diimide (PTCDI)	BASF
ITO covered PET foil	Sigma Aldrich/Merck, 639303
Hellmanex III	Sigma Aldrich/Merck, Z805939
OTS (Trimethoxy(octadecyl)silane)	Sigma Aldrich/Merck, 376213
Acetone	Sigma Aldrich/Merck, 270725
2-propanol	Sigma Aldrich/Merck, 34863-1L
Devices:	
Thermal evaporator for metals	Edwards, model 306A
Thermal evaporator for organics	HHV, model Auto306
Plasma cleaner	Diener, Zepto-RIE
**OEPCs fabrication - *in vivo* application**	
Materials:	
Parylene C	SCS Coatings
MICRO-90® Concentrated Cleaning Solution	International Products Corporation
Phthalocyanine (H_2Pc)	Sigma Aldrich/Merck, 931985
N,N'-dimethyl-3,4,9,10-perylenetetracarboxylic diimide (PTCDI)	BASF
Devices:	
Magentron	Bestec GmbH
Nano Plasma Cleaner	Diener
PlasmaPro 80 RIE	Oxford Instruments
Edwards E306A Thermal Evaporator	Edwards
**Cell culture and cytotoxicity**	
Materials:	
Fisherbrand Sterile Cell Strainers	Fisher Scientific, Cat. No. 22-363-548
Accutase solution	Sigma Aldrich, Cat. No. A6964

Gibco TrypLE Express	Thermo Fisher Scientific, Cat. No. 12605010
Gibco Poly-D-lysine	Thermo Fisher Scientific, Cat. No. A3890401
Gibco Dulbeccos's Minimal Essential Media	Thermo Fisher Scientific, Cat. No. 42430025
Gibco Fetal Bovine Serum, heat inactivated	Thermo Fisher Scientific, Cat. No. 10100147
Gibco MEM Non-Essential Amino Acids Solution	Thermo Fisher Scientific, Cat. No. 11140050
Gibco Penicillin-Streptomycin (10 000 U/mL)	Thermo Fisher Scientific, Cat. No. 15140122
Gibco Neurobasal A	Thermo Fisher Scientific, Cat. No. 10888022
Gibco B-27	Thermo Fisher Scientific, Cat. No. 17504044
Gibco GlutaMAX	Thermo Fisher Scientific, Cat. No. 35050061
Normocin	InvivoGen, Cat. No. ant-nr-1
bFGF	PeproTech, Cat. No. 100-18B-50UG
EGF	PeproTech, Cat. No. AF-100-15-100UG
D-(+)-glucose	Sigma Aldrich, Cat. No. G8270-100G
CyQUANT LDH Cytotoxicity Assay	Thermo Fisher Scientific, Cat. No. C20301
Devices:	
Tissue chopper	McIlwain Tissue Chopper (RRID:SCR_015798)
CASY Cell Counter and Analyser System Model TT	OMNI Life Science (RRID:SCR_002080)
SPECTROstar Omega Microplate Reader	BMG Labtech
**Surgery**	
Materials:	
Fentanyl (Fentanyl-hameln, 50 µg/ml)	Hameln Pharma
Midazolam (Midazolam-hameln, 5 mg/ml)	Hameln Pharma
Medetomidine (Domitor, 1 mg/ml)	Orion Pharma
Flumazenil (Flumazenil Kabi, 0,1 mg/ml)	Fresenius Kabi
Atipamezole (Antisedan, 5 mg/ml)	Orion Pharma
Enrofloxacin (Baytril, 50 mg/ml)	Bayer
Carprofen (Rimadyl, 50 mg/ml)	Pfizer
Natrium chloride 0.9%	Fresenius Kabi
BioMed Clear Resin	Formlabs
iCEM Self Adhesive composite	Heraeus Kulzer
Vicryl Plus, FS-2, 5/0	Ethicon, Cat. No. VCP391H
Trephine drill bits, 10mm length, RA L, 229	Meisinger, Cat. No. 3300229205040
Devices:	
Stereotactic frame	Kopf Instruments
Dental Wireless LED Curing Light	Heraeus Kulzer
**Light stimulation**	
Devices:	

High-Power 1-Channel LED Driver with Pulse Modulation	ThorLabs, Cat. No. DC 2200
**Histology and immunohistochemistry**	
Materials:	
Formaldehyde 37%	Roth, Cat. No. 7398.4
ROTI PreMix PBS	Roth, Cat. No. 0890.2
Triton X-100	Millipore, Cat. No. 1.08603.1000
Sodium citrate tribasic dihydrate	Sigma Aldrich. Cat. No. C7254-1KG
Ethanol absolute 99.9% vol.	AustrAlco
Xylene	Roth, Cat. No. 4436.2
Methanol	VWR internation, Cat. No. NC1179385
Hydrogen peroxide solution 30% (w/w)	Sigma Aldrich, Cat. No. 31642-500ML-M
Normal goat serum	Abcam, Cat. No. ab7481
Gibco Horse serum, heat inactivated	Thermo Fisher Scientific, Cat. No. 26050-088
VECTASTAIN ABC HRP Kit	Vector, Cat. No. PK-4000
ImmPACT DAB Substrate Kit	Vector, Cat. No. SK-4105
Hämalaunlösung n. Mayer	Gatt-Koller, Cat. No. 401296170
Tissue-Tek Glas Mounting Medium	Sakura Finetek, Cat. No. 6419
Fluoroshield with DAPI	Sigma Aldrich, Cat. No. F6057-20ML
Devices:	
Tissue-Tek VIP 5 Tissue Processor	Sakura
TES Valida Modular Paraffin Embedding Center	MEDITE Medical GmbH
Microm HM 560 Cryostat	Thermo Fisher Scientific
Rotary microtome HM355 S	Thermo Fisher Scientific
Decloaking Chamber NxGen	BioCare Medical, Cat. No. DC 2012
Aperio ScanScope AT	Leica
Nikon A1R Confocal Laser Microscope	Nikon (RRID:SCR_020317)
Software:	
Fiji	U. S. National Institutes of Health; Bethesda, Maryland, USA (RRID: SCR_002285)
QuPath (v0.4.3)	RRID:SCR_018257
**SEM preparation and imaging**	
Materials:	
Sodium cacodylate trihydrate	Merck
OsO_4 stock solution(4% in water)	EMS
L(+)-Ascorbic acid	Roth
Ethanol	Lactan

Hexamethyldisilazane	VWR Chemicals
Liquid conductive silver	EMS
Devices:	
Sputter coater SCD500	Bal-Tec
Scanning Electron Microscope Sigma 500 VP	Zeiss
Software:	
ImageSP	SysProg

Table S2. Experimental design and animal number for *in vivo* OEPC implantation and stimulation.

Experiment-No.	Animal age (weeks)	Dura removal	Latency to stimulation	Stimulation (n)	Sham (n)	Light control (n)	Sum
1	12	+	none	3	2	0	5
2	12	-	24 h	3	2	0	5
3	14	+	24 h	3	3	0	6
4	11	+	48 h	3	3	1	7
5	10	+	3 weeks	6	5	7	18
			**Sum**	18	15	8	41

Table S3. List of antigens, applications and dilutions of antibodies used in the *in vitro* and *in vivo* experiments. DAB – 3,3'-Diaminobenzidine; ICC – immunocytochemistry; IF – immunofluorescence; IHC – immunohistochemistry.

Antibody	Host	Application	Dilution	Company	Catalog-No.	# RRID
c-Fos	rabbit	IF ICC	1:1000	Abcam	ab190289	AB_2737414
c-Fos [EPR24046-20]	rabbit	DAB IHC<br>IF IHC	1:100	Abcam	ab289723	-
GFAP [GA5]	mouse	DAB IHC<br>IF IHC	1:500	Thermo Fisher Scientific	14-9892-82	AB_10598206
Iba1	rabbit	DAB IHC	1:2000	FUJIFILM Wako Shibayagi	019-19741	AB_839504
CD45	mouse	DAB IHC	1:50	BD Biosciences	554875	AB_395568
CD68 [ED1]	mouse	DAB IHC	1:50	Origene	BM4000S	AB_1613055
NeuN [A60]	mouse	IF IHC	1:500	Millipore	MAB377	AB_2298772
Calretinin [6A9]	mouse	IF IHC	1:1000	Abcam	ab277631	-
Parvalbumin	chicken	IF IHC	1:1000	Thermo Fisher Scientific	PA5-143579	AB_2942808
Biotinylated anti-rabbit IgG	goat	DAB IHC	1:200-1:400	Vector	BA-1000	AB_2313606
Biotinylated anti-mouse IgG	horse	DAB IHC	1:50-1:200	Vector	BA-2001	AB_2336180
Alexa Fluor™ 488 conjugated anti-rabbit IgG	goat	IF IHC<br>IF ICC	1:500	Thermo Fisher Scientific	A-11008	AB_143165
Alexa Fluor™ 568 conjugated anti-mouse IgG	goat	IF IHC	1:500	Thermo Fisher Scientific	A-11004	AB_2534072
Alexa Fluor™ 647 conjugated anti-chicken IgY	goat	IF IHC	1:500	Thermo Fisher Scientific	A-21449	AB_2535866

Table S4. List of statistical tests and, if applicaple, post hoc tests used in the analysis in the *in vitro* and *in vivo* experiments.

Experiment	Test	Post hoc
LDH – comparisons between groups and time points	Two-way repeated measures ANOVA	Tukey's HSD
LDH – comparison between before and after stimulation	Student's t-test	-
Body mass	Two-way repeated measures ANOVA	-
Nest score	Two-way repeated measures ANOVA	-
c-Fos (+) cell percentage – ITO-OEPC	Kruskal-Wallis test	Dunn's test
c-Fos (+) cell percentage – back electrode – experiment 1 with Au-OEPC	Mann-Whitney U test	-
c-Fos (+) cell percentage – p-n layer (inner and outer parts) – experiment 1 with Au-OEPC	Two-way ANOVA	-
c-Fos (+) cell percentage – back electrode – experiment 2 with Au-OEPC	Student's t-test	-
c-Fos (+) cell percentage – p-n layer – experiment 2 with Au-OEPC	Mann-Whitney U test	
c-Fos (+) cell percentage in brain slices	Two-way ANOVA	Tukey's HSD
